# Second-generation downscaled earth system model data using generative machine learning

**DOI:** 10.1016/j.dib.2025.111774

**Published:** 2025-06-10

**Authors:** Grant Buster, Brandon N. Benton, Deeksha Rastogi, Shih-Chieh Kao, Guilherme Castelao, Jordan Eisenman

**Affiliations:** a Strategic Energy Analysis Center, National Renewable Energy Laboratory, 15013 Denver West Parkway, Golden, CO, 80401, USA; b Computational Sciences and Engineering Division, Oak Ridge National Laboratory, P.O. Box 2008, Oak Ridge, TN, 37831, USA; c Environmental Sciences Division, Oak Ridge National Laboratory, P.O. Box 2008, Oak Ridge, TN, 37831, USA

**Keywords:** Meteorological data, Severe weather, Energy system modelling

## Abstract

The second-generation Sup3rCC dataset provides high-resolution meteorological data generated through the downscaling of multiple earth system models (ESMs) from the Coupled Model Intercomparison Project Phase 6 (CMIP6). This downscaling is performed through application of a generative machine learning approach called Super-Resolution for Renewable Resource Data (sup3r). This dataset builds on the first-generation Sup3rCC data by applying improved bias correction methods and adding downscaled precipitation to the output variables. As with the first Sup3rCC version, the data still include temperature, wind speed and direction at multiple heights, pressure, three components of downwelling solar radiation, and relative humidity—all at 4-kilometer (km) hourly resolution over the contiguous United States. This is a 25x spatial enhancement and 24x temporal enhancement of the source 100-km daily-average ESM data. This extension of the Sup3rCC dataset includes data from six ESMs from two shared socioeconomic pathways (SSPs) totaling 400 years of data with multiple future projections of changing meteorological conditions. The scenario selection was based on a structured evaluation of historical ESM skill and comprehensive representation of possible trajectories of future climate change in temperature, humidity, precipitation, solar irradiance, and near-surface wind speeds. The inclusion of multiple future projections is intended to enable users to assess key drivers of uncertainty and variability. All data are double-bias corrected, resulting in a product that can be used out-of-the-box for energy system analysis with minimal historical bias.

The potential applications of Sup3rCC data extend to various topics in renewable energy resource assessment, energy systems modeling, and grid resilience studies. High-resolution future meteorological projections are critical for evaluating the effects of changing meteorological conditions on renewable energy generation, energy demand, and for optimizing energy storage and grid infrastructure. The 4-km hourly resolution of the downscaled data enables understanding of spatial and temporal variability at the scales necessary for energy system operational planning. In addition, the dataset can support risk assessments with detailed information on possible future extreme weather events and long-term meteorological variability at scales relevant to energy infrastructure. The second-generation Sup3rCC dataset enables more precise modeling of energy resilience and adaptation strategies in response to changing meteorological conditions through an enhanced representation of possible future meteorological conditions.

Specifications Table

**Table:** 

Subject	Earth & Environmental Sciences
Specific subject area	High-resolution meteorological data with future projections for studying renewable energy resources and energy system resilience.
Type of data	Spatiotemporal data files (.h5) Processed
Data collection	The data were collected through the downscaling of earth system model (ESM) data using the previously developed Super-Resolution for Renewable Resource Data (sup3r) generative machine learning approach [1]. Six ESMs were selected for downscaling based on a structured evaluation that assessed model historical skill and spread of future projections [2,3]. ESM data were downscaled 25x and 24x in the spatial and temporal dimensions, respectively, resulting in a final data resolution of 4 km hourly. The generative models used in the downscaling process are included in this data release and were trained using the sup3r software package v0.2.2 [4].
Data source location	Data collected over the contiguous United States from CMIP6 data hosted on the Earth System Grid Federation (https://esgf.github.io/index.html); output data stored in the Open Energy Data Initiative (OEDI, https://data.openei.org/submissions/5839) and Amazon Web Services (AWS, s3://nrel-pds-sup3rcc/)
Data accessibility	Repository name: Sup3rCC Data identification number: https://doi.org/10.25984/1970814 Direct URL to data: https://data.openei.org/submissions/5839 Instructions for accessing the data are included in the data repository.
Related research article	None

## 1 Value of the Data

• High-resolution future meteorological projections are critical for analyzing potential changes in energy generation and demand.
• These data include high-resolution output variables coherent across space, time, and multiple variables that are well suited for large-scale energy system analyses and grid resilience studies.
• These data support risk assessments by providing detailed information on possible future extreme weather events and long-term meteorological variability at spatial and temporal scales relevant to energy infrastructure.
• Double-bias correction ensures the data are representative of the current climate so future projections may be analyzed for change signal with minimal concern of historical bias.
• Uncertainty is communicated via the ensemble of downscaled scenarios, each of which represents a possible trajectory of future climate change based on the latest earth system science.

## 2 Background

According to data from the U.S. Department of Energy (DOE), 46% of major power outages in the United States from 2000 to 2020 were because of natural or weather-related events, with an increase in the total number of outages over time [5]. Meteorological data and projections of future climate change are vital inputs to energy system planning that can improve grid reliability and resilience [6]. Previous efforts such as Sup3rCC [1] have used generative machine learning to develop high-resolution meteorological datasets with future projections designed specifically for applications in energy systems research. Sup3rCC trains generative super resolution models on historical weather data that can then produce high-resolution weather data, including projections of future change, based on low-resolution forcings from ESMs. The process is computationally efficient and outputs data that are coherent across space, time, and multiple variables [1]. However, the original v0.1.0 Sup3rCC data exhibited substantial bias in the historical climate and had a limited representation of uncertainty with only two scenarios that were chosen without a structured selection process [1]. This manuscript documents the data and validation basis for the second-generation v0.2.2 Sup3rCC dataset that improves upon these two limitations based on the same generative machine learning methods as the original work.

## 3 Data Description

As described in Table 1, the Sup3rCC v0.2.2 data includes six downscaled ESMs using public data retrieved from the CMIP6 archive based on the following selection process. First, all ESMs with variables required by Sup3rCC were systematically evaluated for historical skill and spread of future projection [2]. Second, five ESMs and the SSP2-4.5 scenario were selected for downscaling based on the quantitative evaluation and subsequent discussion with stakeholders [3]. The following five ESMs were selected: TaiESM1 [7], EC-Earth3-CC [8], GFDL-CM4 [9], EC-Earth3-Veg [10], and MPI-ESM1-2-HR [11]. Finally, MRI-ESM2-0 [12] for SSP5-8.5 was added for direct comparison to the original Sup3rCC data release.

**Table 1:** Sup3rCC v0.2.2 scenario names and corresponding source ESMs and scenarios from CMIP6.

Scenario Name	ESM	Scenario	Years
sup3rcc_conus_ecearth3cc_historical_r1i1p1f1	EC-Earth3-CC (r1i1p1f1)	Historical	2000–2014
sup3rcc_conus_ecearth3cc_ssp245_r1i1p1f1	EC-Earth3-CC (r1i1p1f1)	SSP2-4.5	2015–2059
sup3rcc_conus_ecearth3veg_historical_r1i1p1f1	EC-Earth3-Veg (r1i1p1f1)	Historical	2000–2014
sup3rcc_conus_ecearth3veg_ssp245_r1i1p1f1	EC-Earth3-Veg (r1i1p1f1)	SSP2-4.5	2015–2059
sup3rcc_conus_gfdlcm4_historical_r1i1p1f1	GFDL-CM4 (r1i1p1f1)	Historical	2000–2014
sup3rcc_conus_gfdlcm4_ssp245_r1i1p1f1	GFDL-CM4 (r1i1p1f1)	SSP2-4.5	2015–2059
sup3rcc_conus_mpiesm12hr_historical_r1i1p1f1	MPI-ESM1-2-HR (r1i1p1f1)	Historical	2000–2014
sup3rcc_conus_mpiesm12hr_ssp245_r1i1p1f1	MPI-ESM1-2-HR (r1i1p1f1)	SSP2-4.5	2015–2059
sup3rcc_conus_mriesm20_historical_r1i1p1f1	MRI-ESM2-0 (r1i1p1f1)	Historical	2000–2014
sup3rcc_conus_mriesm20_ssp585_r1i1p1f1	MRI-ESM2-0 (r1i1p1f1)	SSP5-8.5	2015–2059
sup3rcc_conus_taiesm1_historical_r1i1p1f1	TaiESM1 (r1i1p1f1)	Historical	2000–2014
sup3rcc_conus_taiesm1_ssp245_r1i1p1f1	TaiESM1 (r1i1p1f1)	SSP2-4.5	2015–2099

*Note:* “r1i1p1f1” is a variant label from CMIP6 representing different realizations of the same ESM/SSP combination and could be incremented later to further expand the Sup3rCC ensemble.

ESM data are downscaled from 2000 through 2059 to represent projected changes from the current climate through midcentury. Years 2000–2014 are taken from the “historical” CMIP6 scenario whereas years after 2014 are taken from the respective SSP scenarios. Note that none of the historical ESM or Sup3rCC years represents “real-world” historically observed weather on an hourly basis—only the historical climate based on multidecadal statistics. For example, historical events such as Winter Storm Yuri will not be precisely reproduced in the Sup3rCC data, but similar events will be present at different times in different Sup3rCC data years. TaiESM1 was additionally downscaled through 2099 to provide one estimate of future climate change through the end of the twenty-first century.

Data are stored in the Open Energy Data Initiative (OEDI) [13] in files with the name “{scenario_name}_{group}_{year}.h5” where “scenario_name” is specified in Table 1, the “group” is a subset of the Sup3rCC variables specified in Table 2, and “year” is the ESM weather year ranging from 2000 to 2099. The files strive to be self-documenting with file attributes specifying dataset names, scale factors, units, and Sup3rCC software job configurations. In total, there are 13 output variables described in Table 2 representing key meteorological variables that affect energy generation and demand. More detailed modeling activities may require additional meteorological inputs such as 3D wind fields around a turbine, but here we prioritize variables commonly used in bulk energy system modeling.

**Table 2:** Sup3rCC variable names, descriptions, file tags, units, and spatiotemporal resolutions.

Variable Name	Description	Group	Units	Resolution
meta	Geospatial meta data describing the spatial dimension of the data	N/A^a^	Varying	4-km
time_index	Time index recording instantaneous timestamps for the temporal dimension of the data	N/A^a^	Universal time coordinated (UTC)	hourly
temperature_2m	Dry-bulb air temperature 2 meters (m) above the surface	trh	Degrees Celsius	4-km hourly
relativehumidity_2m	Relative humidity 2 m above the surface	trh	Percentage	4-km hourly
windspeed_10m	Horizontal wind speed 10 m above the surface	wind	m/s	4-km hourly
windspeed_100m	Horizontal wind speed 100 m above the surface	wind	m/s	4-km hourly
windspeed_200m	Horizontal wind speed 200 m above the surface	wind	m/s	4-km hourly
winddirection_10m	Direction from which wind is blowing 10 m above the surface	wind	Degrees clockwise from north	4-km hourly
winddirection_100m	Direction from which wind is blowing 100 m above the surface	wind	Degrees clockwise from north	4-km hourly
winddirection_200m	Direction from which wind is blowing 200 m above the surface	wind	Degrees clockwise from north	4-km hourly
dni	Direct normal irradiance	solar	Watts per square meter (W/m2)	4-km hourly
ghi	Global horizontal irradiance	solar	W/m2	4-km hourly
dhi	Diffuse horizontal irradiance	solar	W/m2	4-km hourly
pr	Daily average precipitation flux	pr	Kilograms per square meter per second (kg/m2s)	4-km daily
pressure_0m	Air pressure at the surface	pressure	Pascals (Pa)	4-km hourly

a The “meta” and “time_index” fields are present in every file across all variable groups.

The following figures present characteristics of the new v0.2.2 Sup3rCC data and reproduce the previous validation basis in comparison to the original v0.1.0 data. Fig. 1, Fig. 2, Fig. 3, Fig. 4, Fig. 5 present historical bias in one of the v0.2.2 Sup3rCC downscaled ESMs versus the high-resolution historical weather datasets documented in Table 3. Fig. 6, Fig. 7, Fig. 8, Fig. 9, Fig. 10 present historical maps of key variables from the new v0.2.2 Sup3rCC SSP2-4.5 scenarios, including projections of change through mid-century (future conditions minus historical conditions). Fig. 11, Fig. 12 show comparisons of Sup3rCC data to the raw and bias-corrected coarse ESM data to illustrate the effects of the new double-bias correction process. Fig. 13, Fig. 14, Fig. 15, Fig. 16, Fig. 17, Fig. 18, Fig. 19, Fig. 20, Fig. 21, Fig. 22, Fig. 23, Fig. 24, Fig. 25, Fig. 26, Fig. 27, Fig. 28, Fig. 29, Fig. 30, Fig. 31, Fig. 32, Fig. 33, Fig. 34, Fig. 35 reproduce the previous Sup3rCC validation basis [1] while comparing data versions to historical datasets for spatial, temporal, and statistical data attributes.

**Fig. 1:**
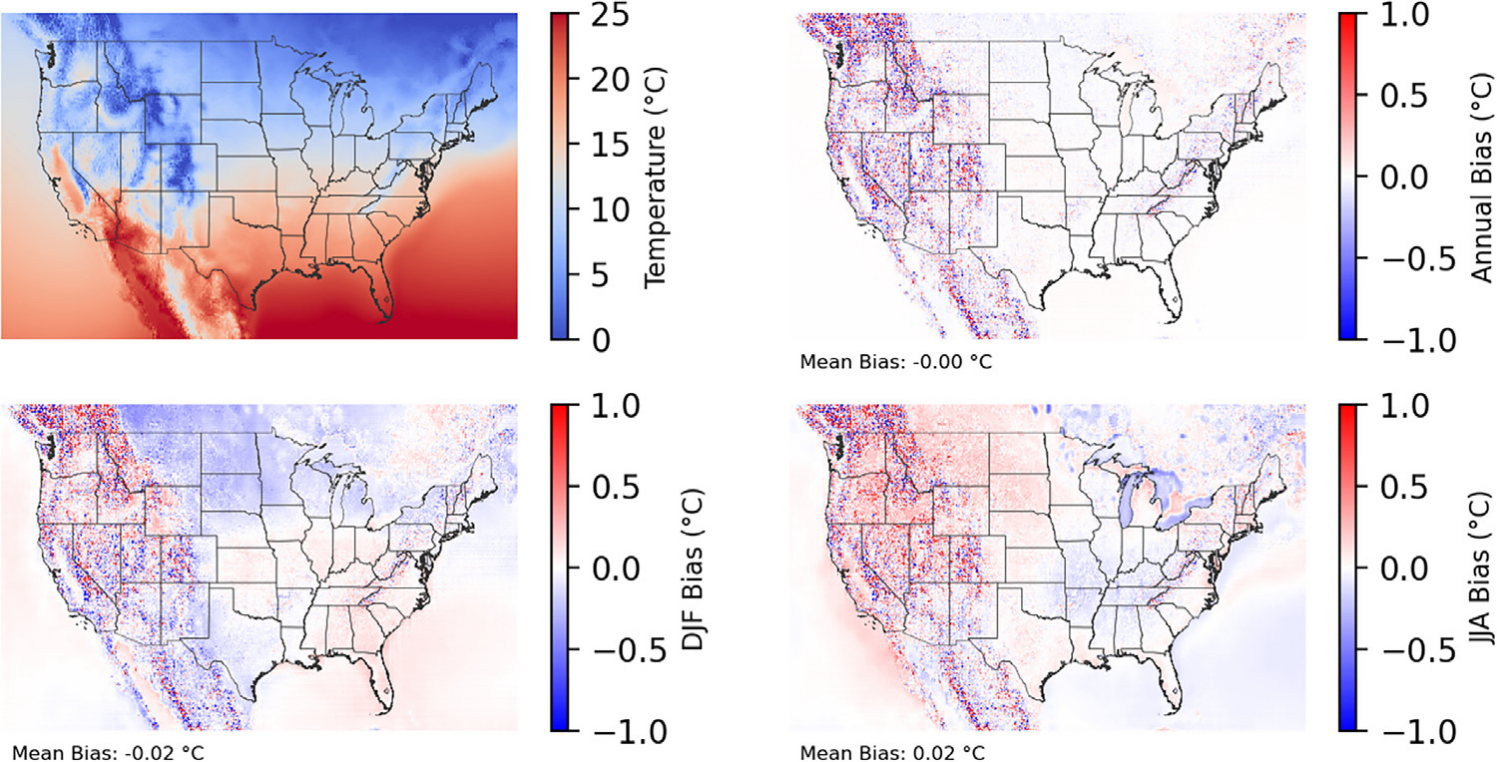
Multiyear mean and bias maps for dry-bulb air temperature for v0.2.2 Sup3rCC-TaiESM1 over the overlapping historical years 1980–2019. Bias is calculated as Sup3rCC minus data from ERA5 interpolated to 4 km with elevation adjustment. Top left panel is Sup3rCC mean values; top right is mean annual bias vs. ERA5; bottom left is mean bias for December, January, and February (DJF); bottom right is mean bias for June, July, and August (JJA).

**Fig. 2:**
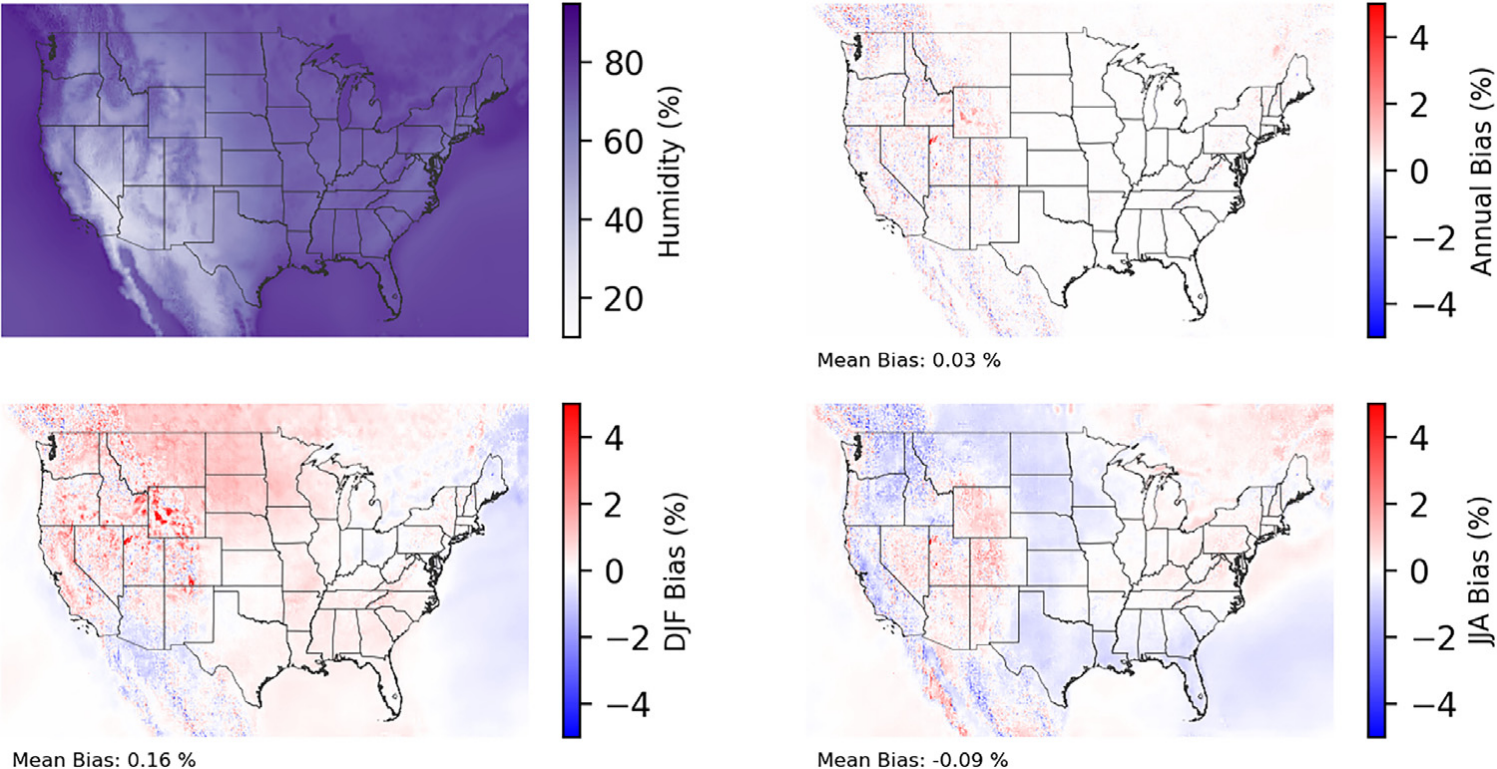
Multiyear mean and bias maps for relative humidity for v0.2.2 Sup3rCC-TaiESM1 over the overlapping historical years 1980–2019. Bias is calculated as Sup3rCC minus data from ERA5 interpolated to 4 km with elevation adjustment. Top left panel is Sup3rCC mean values; top right is mean annual bias vs. ERA5; bottom left is mean bias for December, January, and February (DJF); bottom right is mean bias for June, July, and August (JJA).

**Fig. 3:**
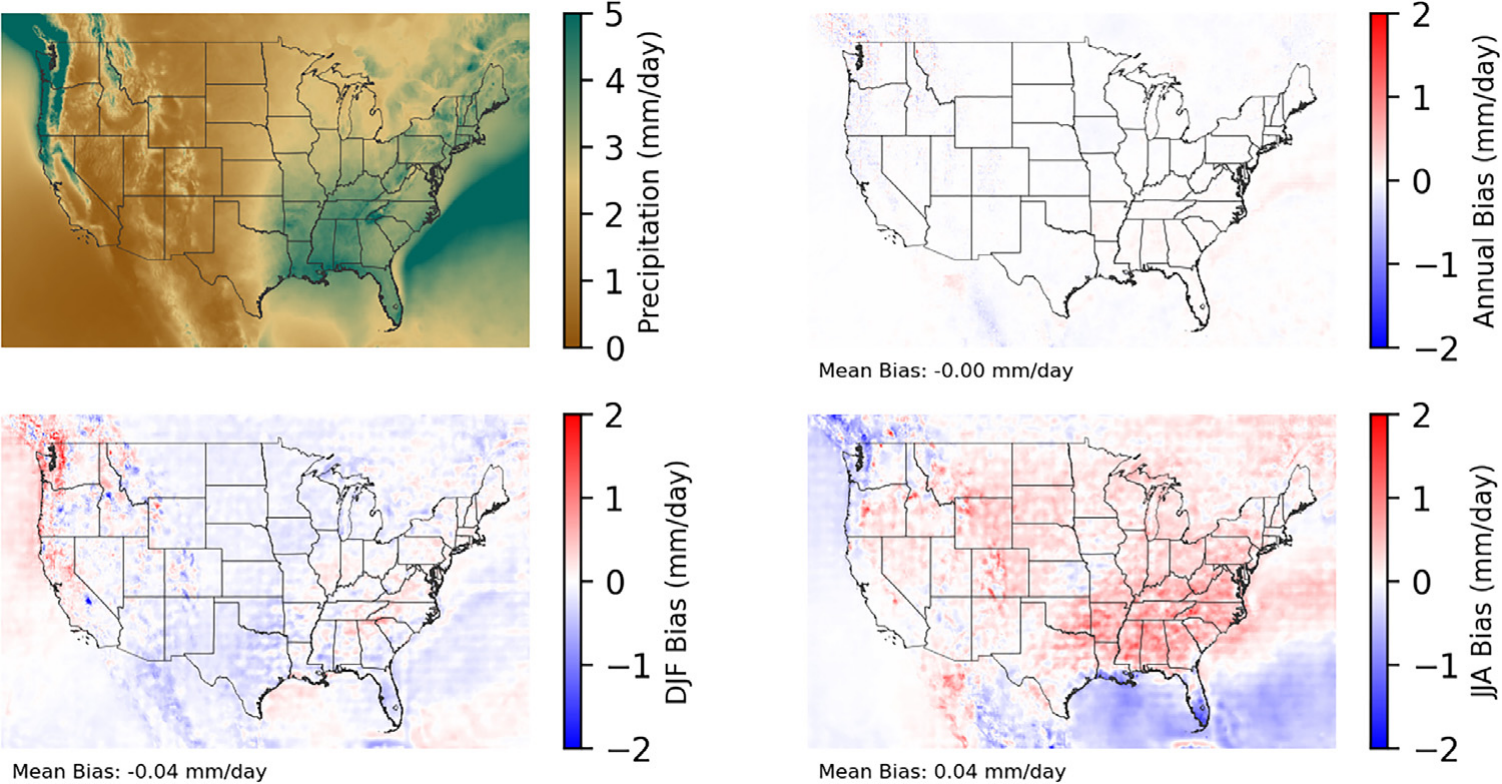
Multiyear mean and bias maps for precipitation for v0.2.2 Sup3rCC-TaiESM1 over the overlapping historical years 1980–2019. Bias is calculated as Sup3rCC minus data from Daymet. Top left panel is Sup3rCC mean values; top right is mean annual bias vs. Daymet; bottom left is mean bias for December, January, and February (DJF); bottom right is mean bias for June, July, and August (JJA).

**Fig. 4:**
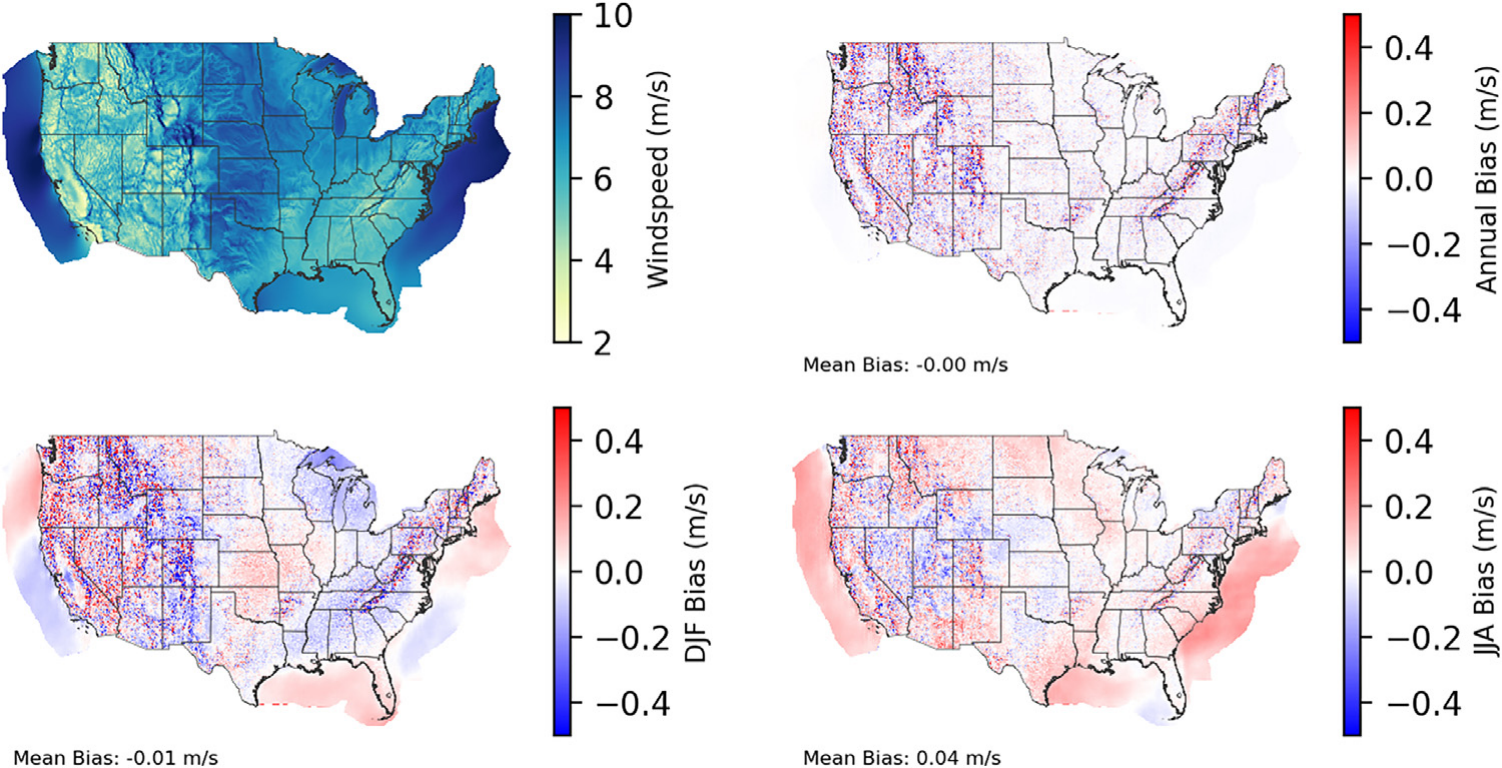
Multiyear mean and bias maps for 100-m wind speed for v0.2.2 Sup3rCC-TaiESM1 over the overlapping historical years. Bias is calculated as Sup3rCC (2000–2019) minus data from the Wind Integration National Dataset Toolkit (WTK) and High-Resolution Rapid Refresh (HRRR) models (2007–2024 without 2014). Top left panel is Sup3rCC mean values; top right is mean annual bias vs. WTK and HRRR; bottom left is mean bias for December, January, and February (DJF); bottom right is mean bias for June, July, and August (JJA).

**Fig. 5:**
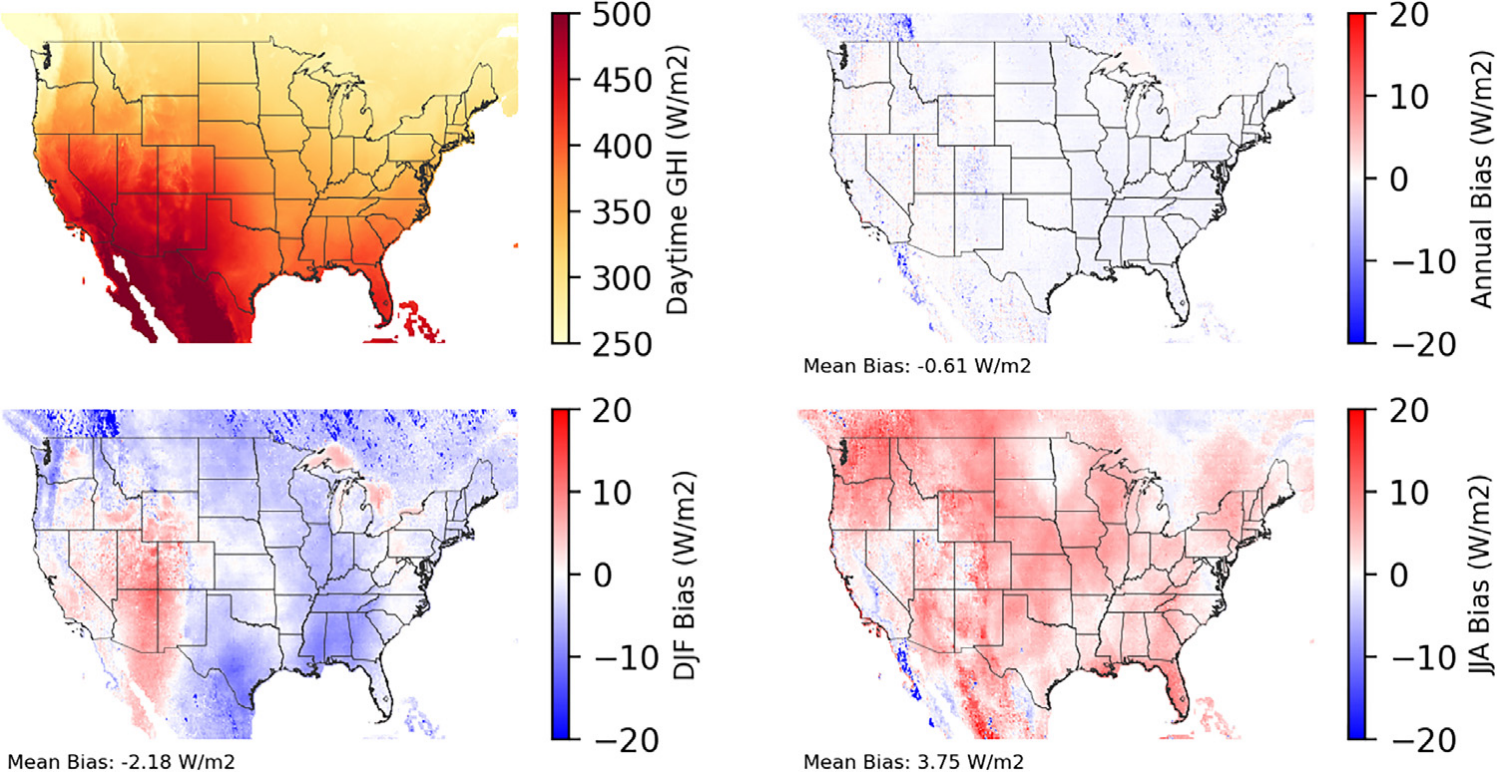
Multiyear mean and bias maps for daytime global horizontal irradiance (GHI) for v0.2.2 Sup3rCC-TaiESM1 over the overlapping historical years 2000–2019. Bias is calculated as Sup3rCC minus data from the National Solar Radiation Database (NSRDB). Total annual GHI and bias values including nighttime hours will be approximately ½ of the values shown here. Top left panel is Sup3rCC mean values; top right is mean annual bias vs. NSRDB; bottom left is mean bias for December, January, and February (DJF); bottom right is mean bias for June, July, and August (JJA).

**Table 3:** Specifications for Sup3rCC v0.2.2 double-bias correction.

Bias-Correction Resolution	Temporal Extent	Variable	Historical Data Source	Bias Correction Equation
100-km Daily	1980–2019	Temperature	ERA5 [16]	Equation 2
100-km Daily	1980–2019	Relative Humidity	ERA5 [16]	Equation 1
100-km Daily	1980–2019	Precipitation	Daymet [17]	Equation 1
100-km Daily	1980–2019	Windspeed (vectors)	ERA5 [16]	Equation 2
100-km Daily	2000–2019	Irradiance	NSRDB [20]	Equation 1
4-km Hourly	1980–2019	Temperate	ERA5^a^ [16]	Equation 2
4-km Hourly	1980–2019	Relative Humidity	ERA5^a^ [16]	Equation 1
4-km Daily	1980–2019	Precipitation	Daymet [17]	Equation 1
4-km Hourly	2007–2024^b^	Windspeed (scalars)	WTK [18]+HRRR [19]	Equation 1
4-km Hourly	2000–2019	Irradiance	NSRDB [20]	Equation 1
4-km Hourly	2007–2024^b^	Surface Pressure	WTK [18]+HRRR [19]	N/A^c^

a 31-km hourly temperature and relative humidity from ERA5 were interpolated to 4-km hourly using lapse rates and elevation adjustments.

b High-resolution wind data were used from the WTK (2007–2013) and from the HRRR (2015–2024). Native level data from the f02 forecast hours linearly interpolated to the desired height above surface were used from HRRR. Data from 2014 were not available from either dataset.

c Surface pressure is corrected using a simple additive correction factor.

**Fig. 6:**
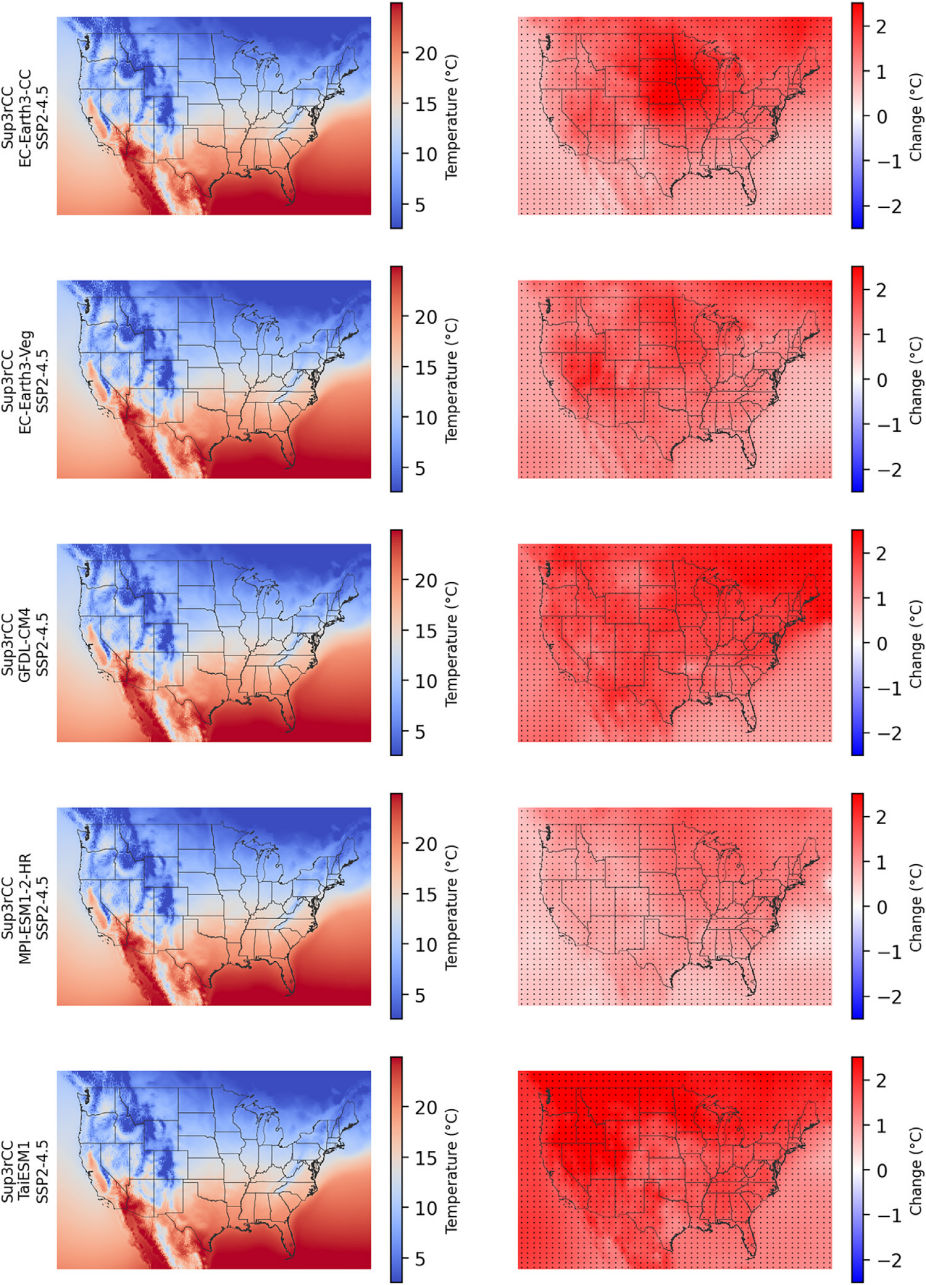
Maps of annual average dry-bulb temperature in the current climate (2000–2019, left) and changes in temperature through midcentury (2040–2059 minus 2000–2019, right) for v0.2.2 Sup3rCC SSP2-4.5 scenarios in each row. The change maps include stippling where four or more of the downscaled data products are in agreement on the sign of change.

**Fig. 7:**
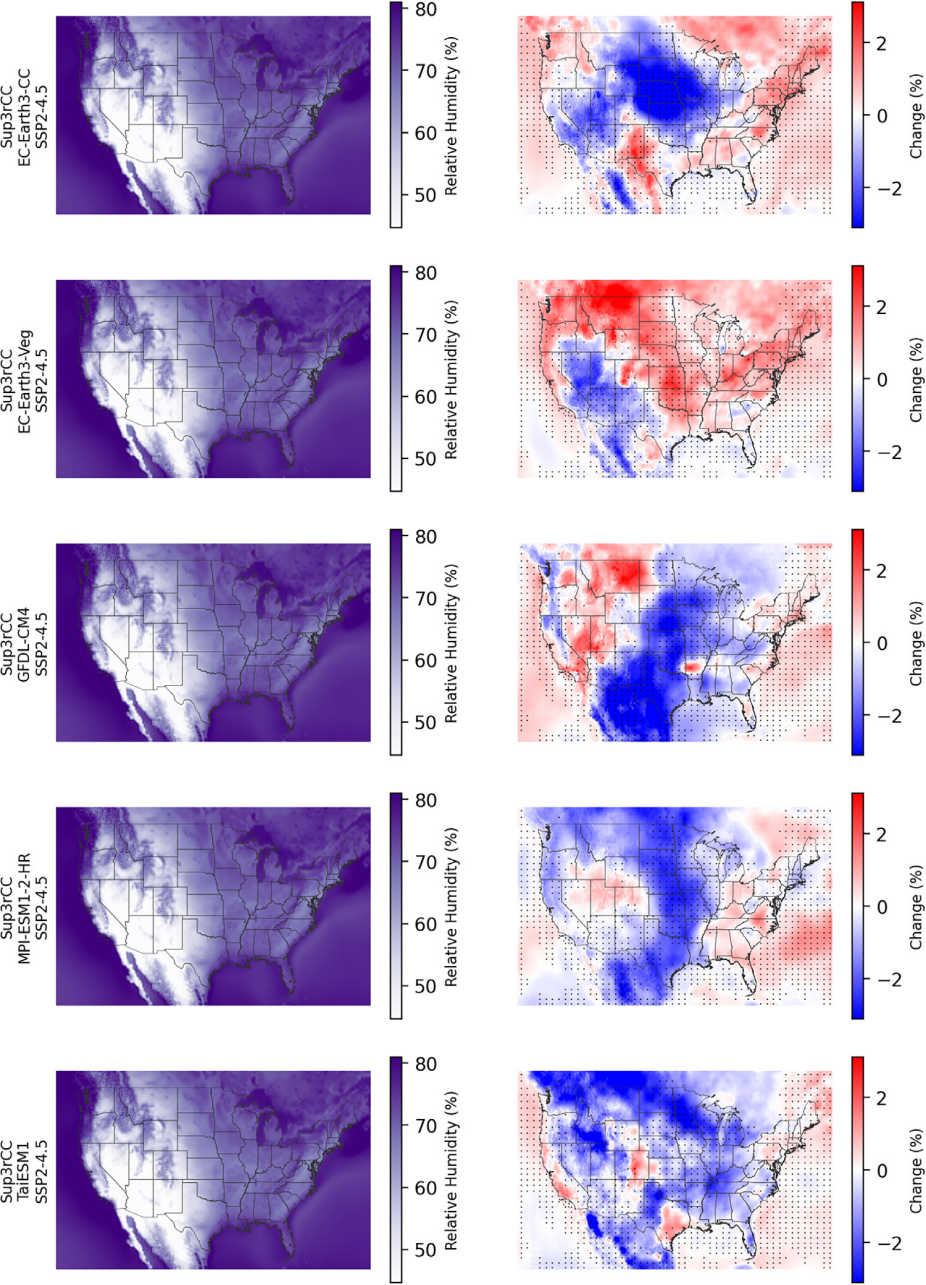
Maps of annual average relative humidity in the current climate (2000–2019, left) and changes in relative humidity through midcentury (2040–2059 minus 2000–2019, right) for v0.2.2 Sup3rCC SSP2-4.5 scenarios in each row. The change maps include stippling where four or more of the downscaled data products are in agreement on the sign of change.

**Fig. 8:**
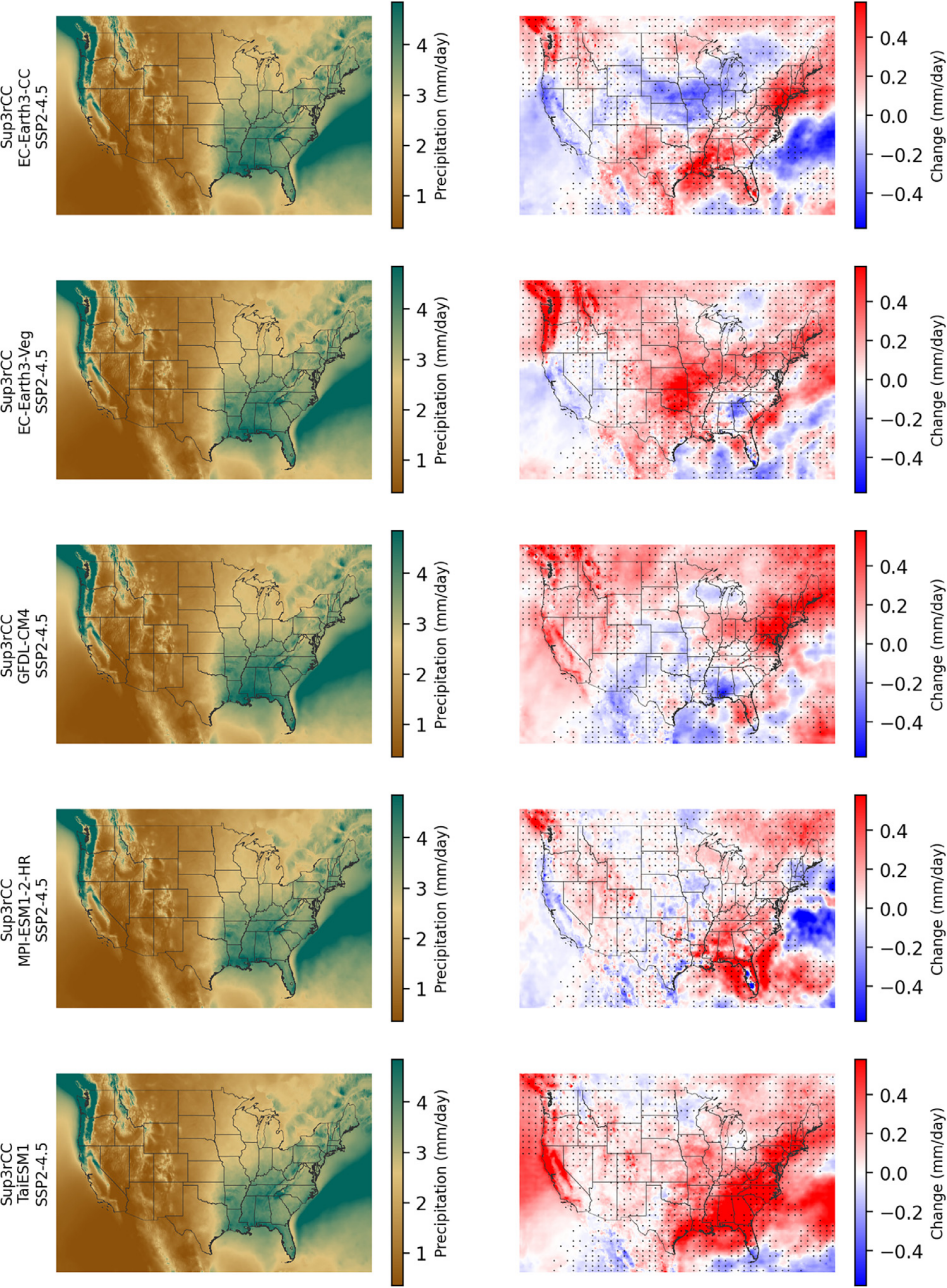
Maps of annual average precipitation in the current climate (2000–2019, left) and changes in precipitation through midcentury (2040–2059 minus 2000–2019, right) for v0.2.2 Sup3rCC SSP2-4.5 scenarios in each row. The change maps include stippling where four or more of the downscaled data products are in agreement on the sign of change.

**Fig. 9:**
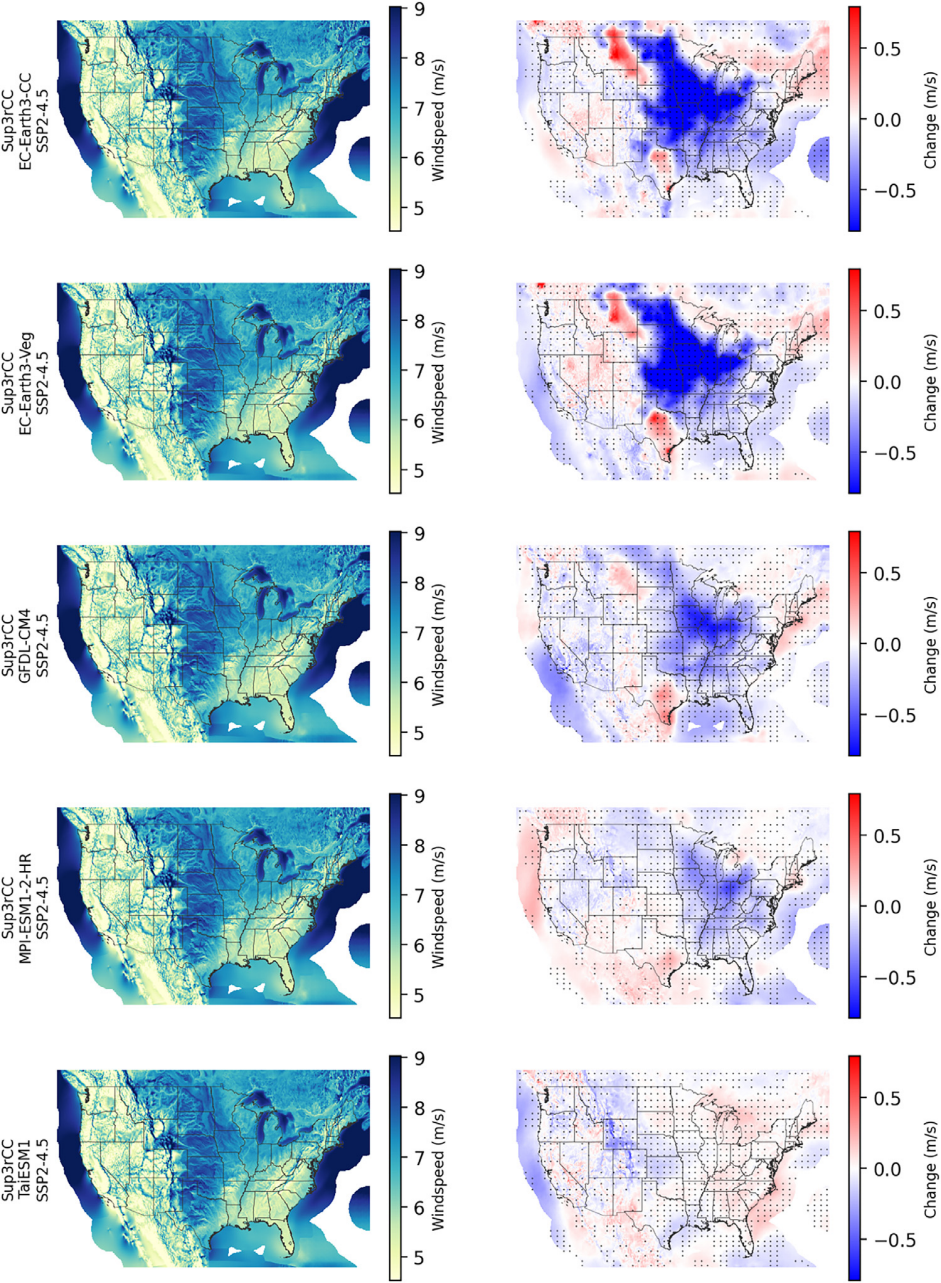
Maps of annual average 100-m wind speed in the current climate (2000–2019, left) and changes in windspeed through midcentury (2040–2059 minus 2000–2019, right) for v0.2.2 Sup3rCC SSP2-4.5 scenarios in each row. The change maps include stippling where four or more of the downscaled data products are in agreement on the sign of change.

**Fig. 10:**
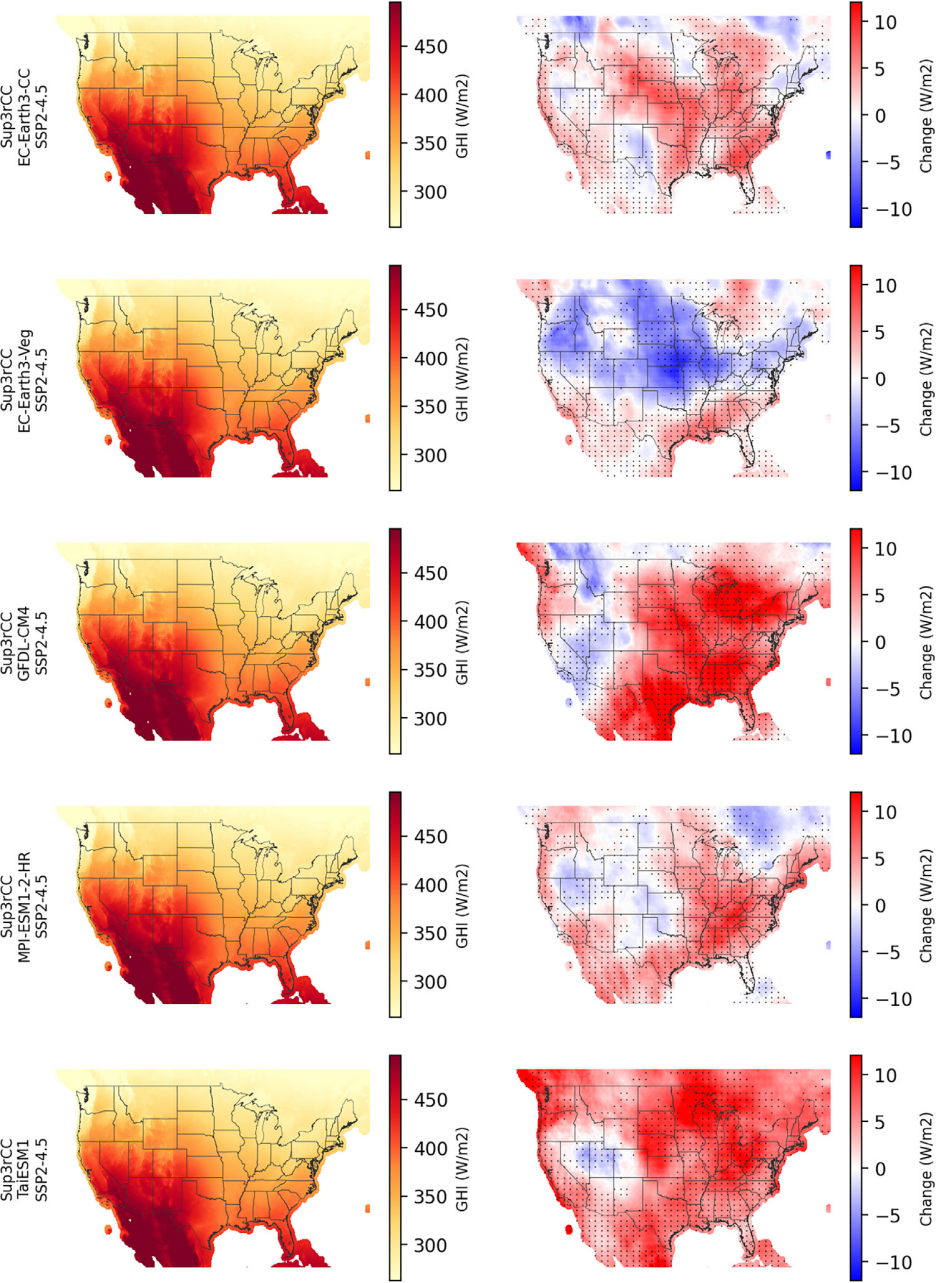
Maps of annual average daytime GHI in the current climate (2000–2019, left) and changes in daytime GHI through mid-century (2040–2059 minus 2000–2019, right) for v0.2.2 Sup3rCC SSP2-4.5 scenarios in each row. The change maps include stippling where four or more of the downscaled data products are in agreement on the sign of change. Total annual GHI and change values will be approximately ½ of the values shown here.

**Fig. 11:**
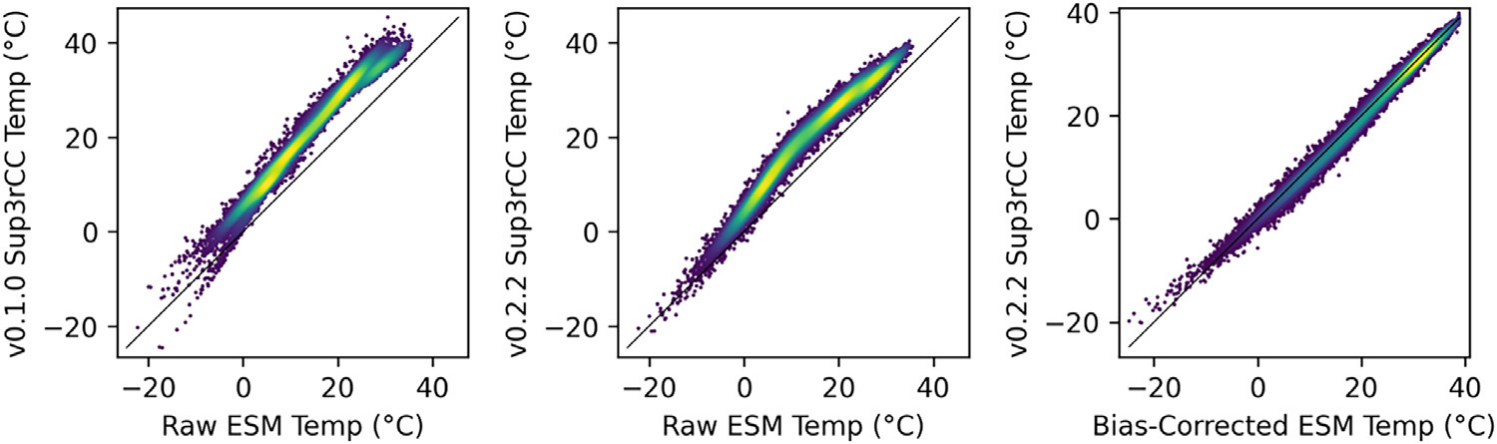
4-km Sup3rCC daily maximum dry-bulb air temperature in Denver, Colorado vs. ESM data for 2000–2059 comparing the original v0.1.0 Sup3rCC data (left), the new v0.2.2 Sup3rCC data (middle), and re-coarsened 100-km v0.2.2 Sup3rCC data vs. the input bias-corrected ESM data (right).

**Fig. 12:**
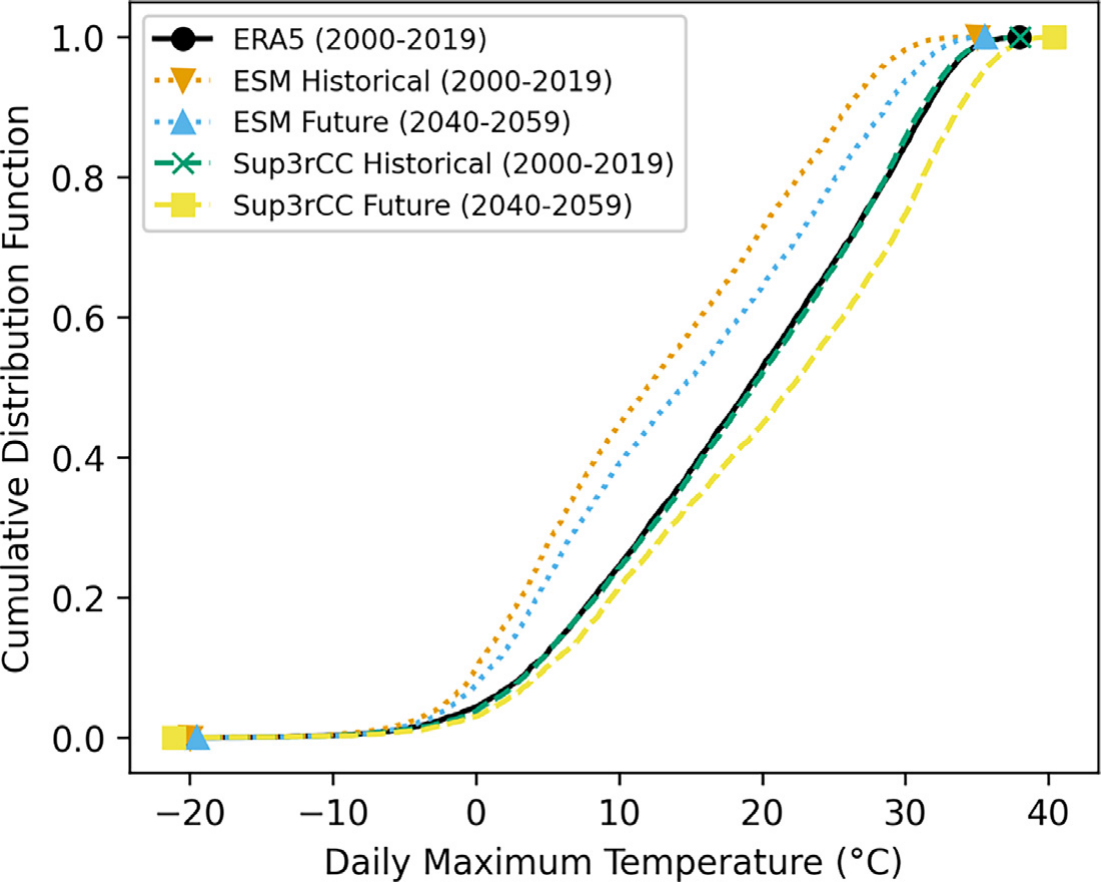
Daily maximum dry-bulb air temperature cumulative distribution functions in Denver, Colorado comparing historical reanalysis from 4-km interpolated ERA5 to raw historical and future ESM data and historical and future data from Sup3rCC v0.2.2.

**Fig. 13:**
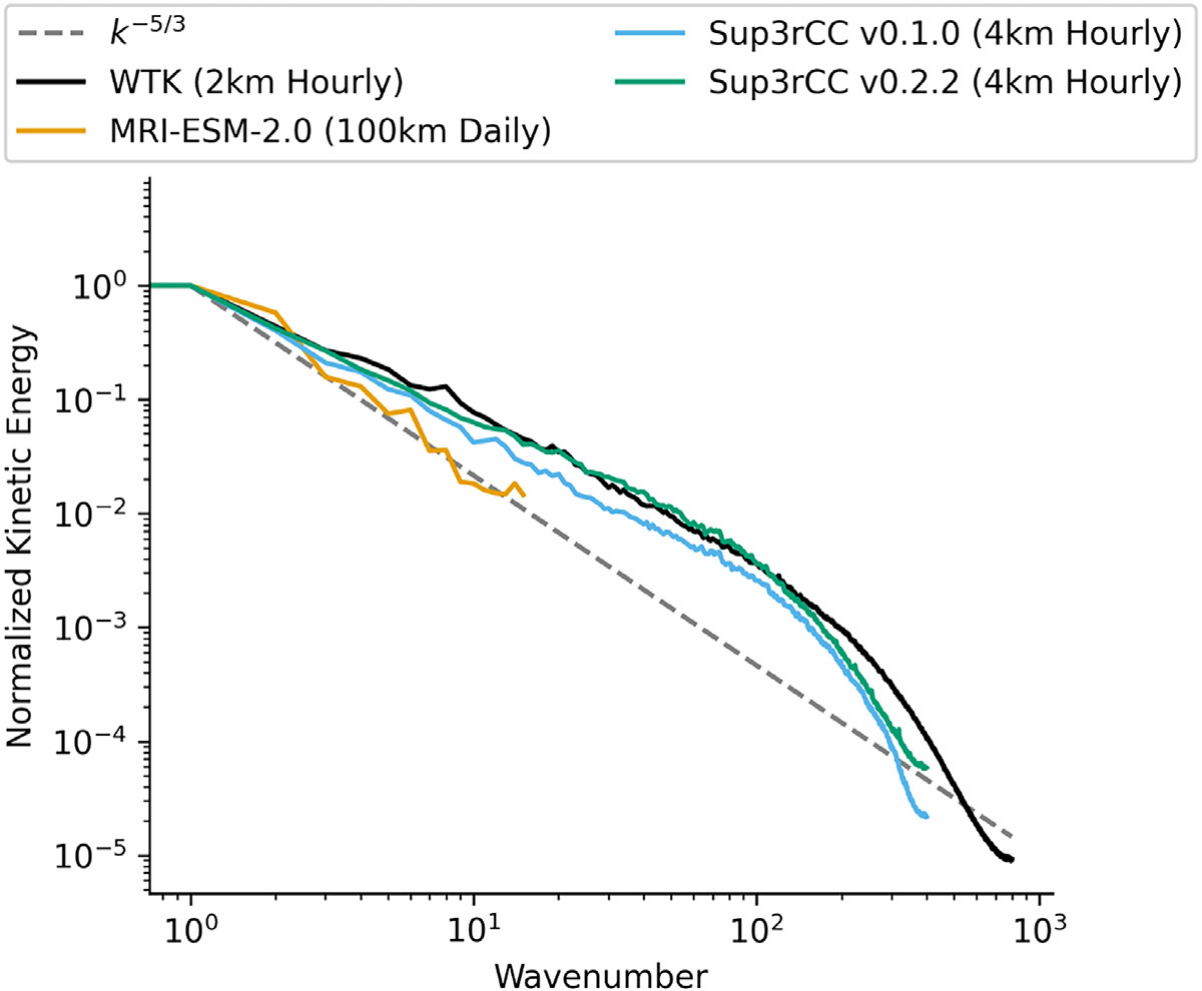
Wind kinetic energy spectra comparing Sup3rCC versions downscaling MRI-ESM-2.0 against the original ESM data and the WTK.

**Fig. 14:**
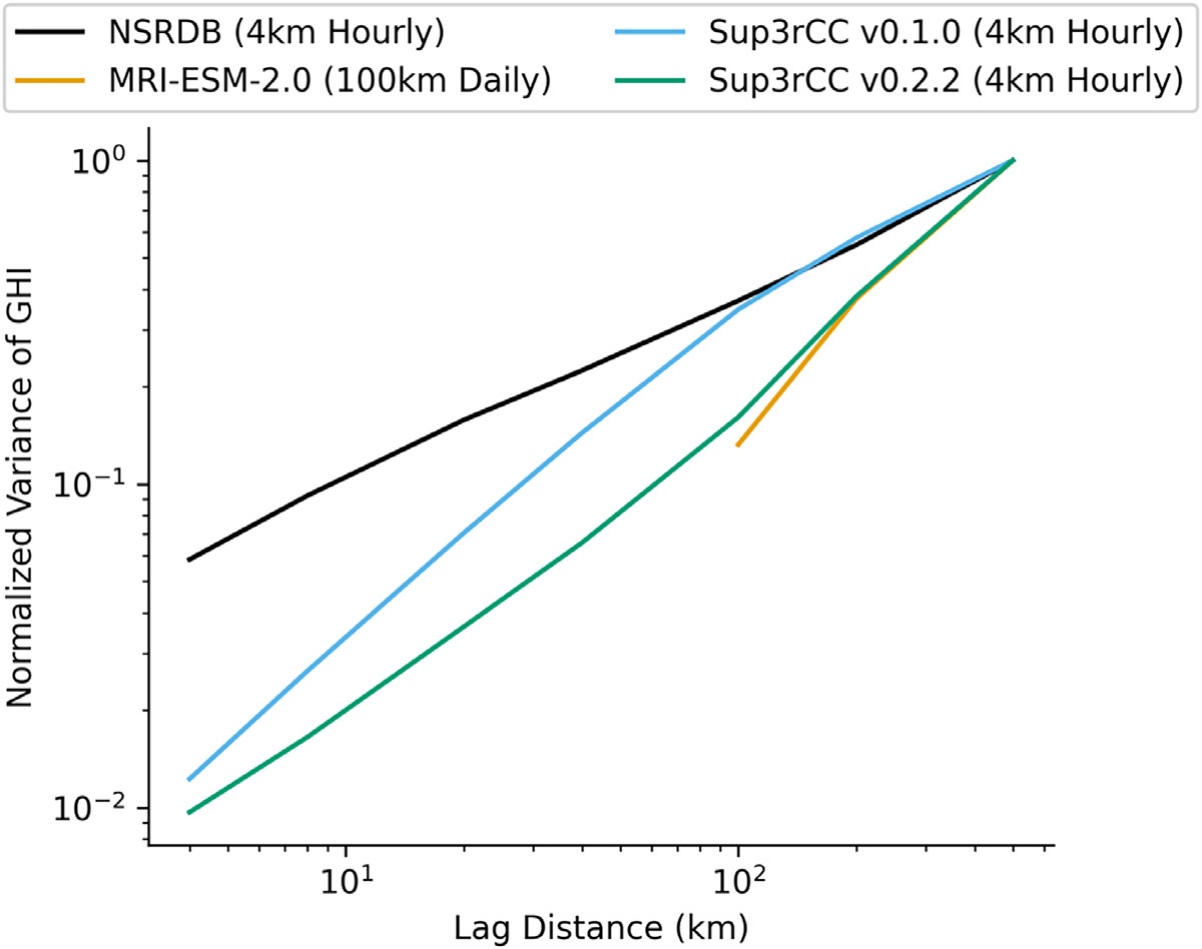
Semivariograms of solar GHI comparing Sup3rCC versions downscaling MRI-ESM-2.0 against the original ESM data and the NSRDB.

**Fig. 15:**
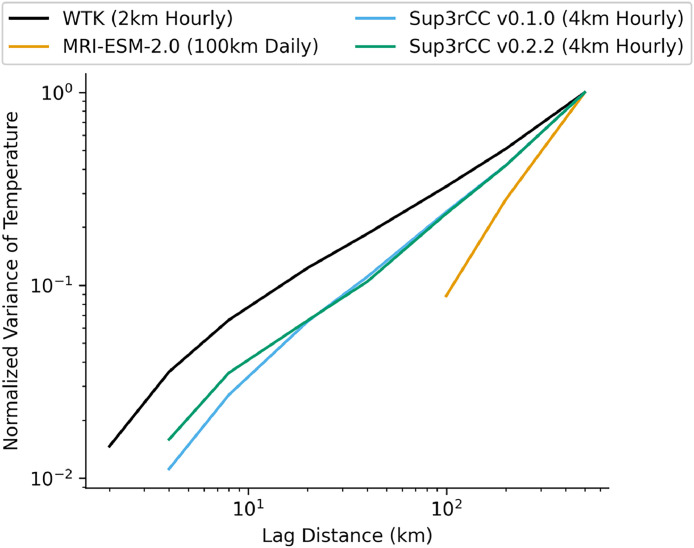
Semivariograms of dry-bulb air temperature comparing Sup3rCC versions downscaling MRI-ESM-2.0 against the original ESM data and the WTK.

**Fig. 16:**
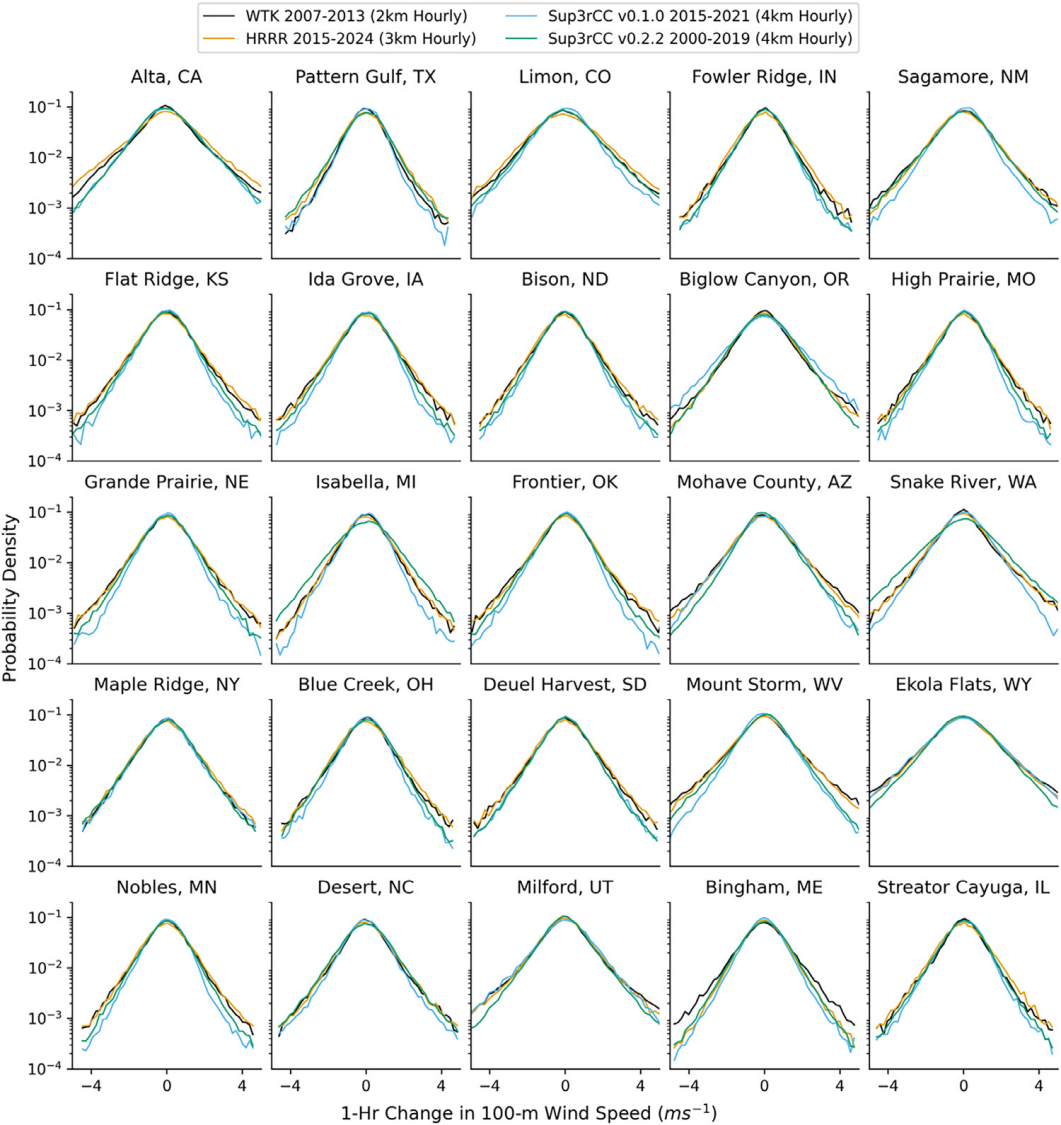
Probability distributions for 1-hour changes in hourly wind speeds. Data are included at 25 large wind energy facilities in the contiguous United States comparing the WTK, HRRR, and Sup3rCC versions at a 100-m hub height.

**Fig. 17:**
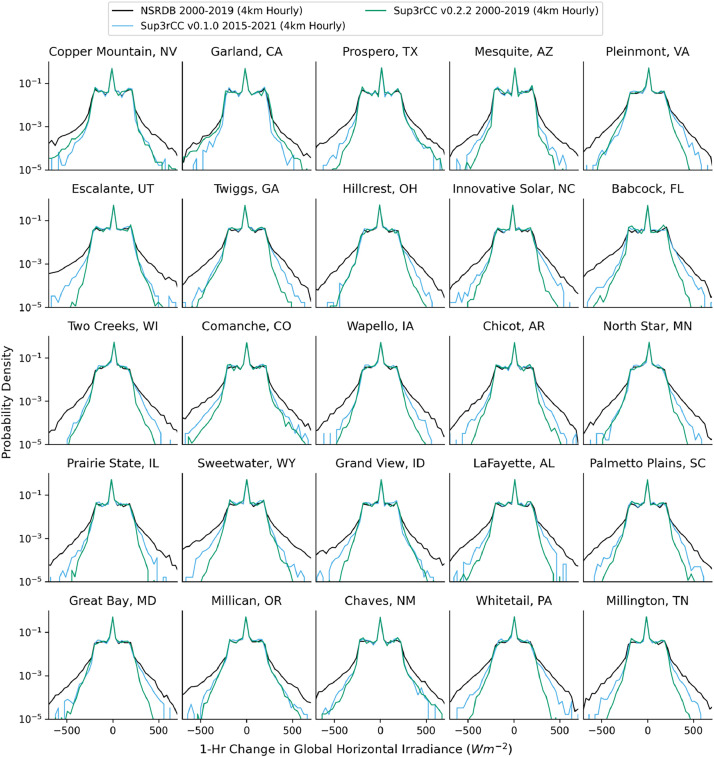
Probability distributions for 1-hour changes in GHI. Data are included for 25 large solar energy facilities in the contiguous United States comparing the NSRDB and Sup3rCC versions. Nighttime values are included.

**Fig. 18:**
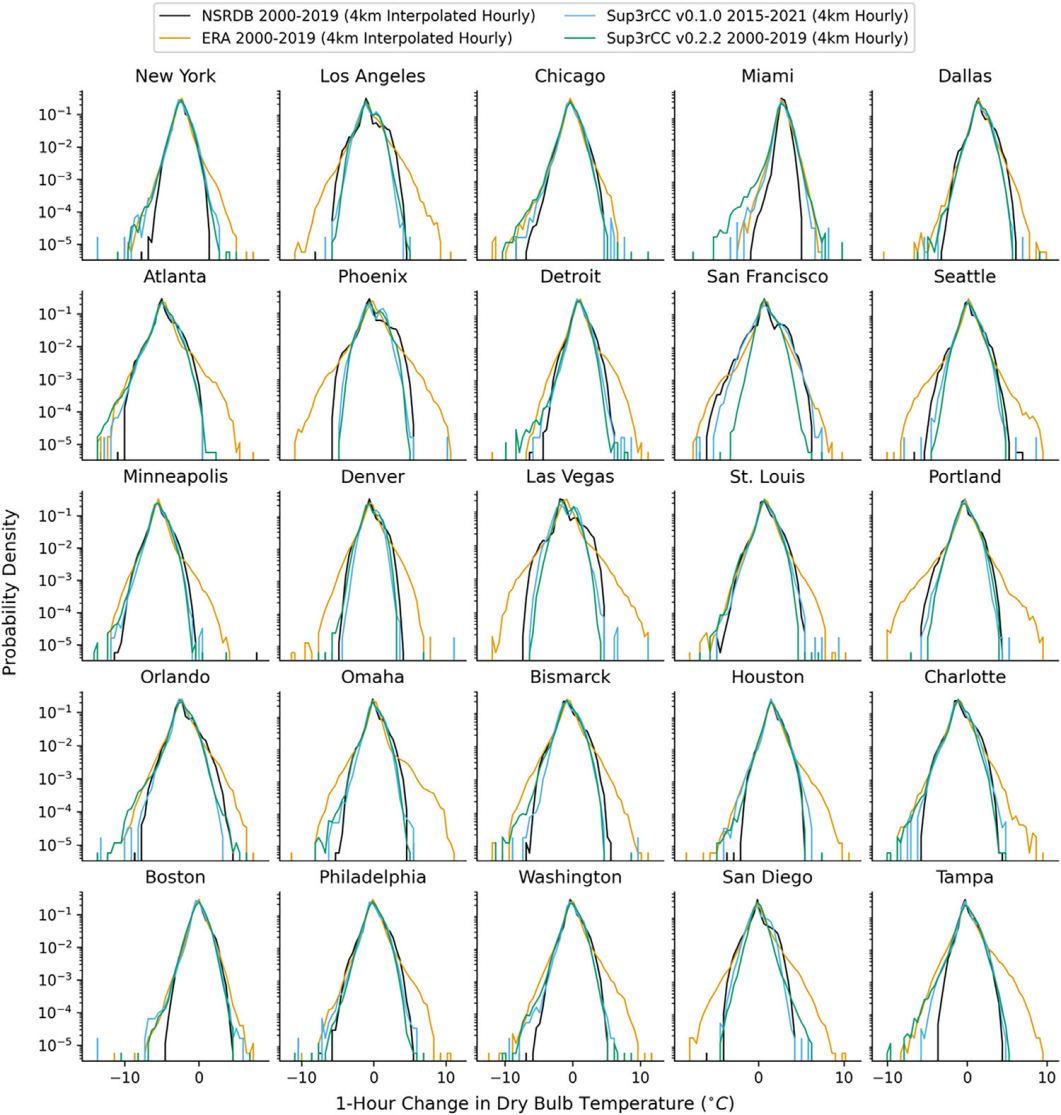
Probability distributions for 1-hour changes in dry-bulb temperature. Data are included for 25 of the largest cities in the contiguous United States comparing the NSRDB, ERA5, and Sup3rCC versions.

**Fig. 19:**
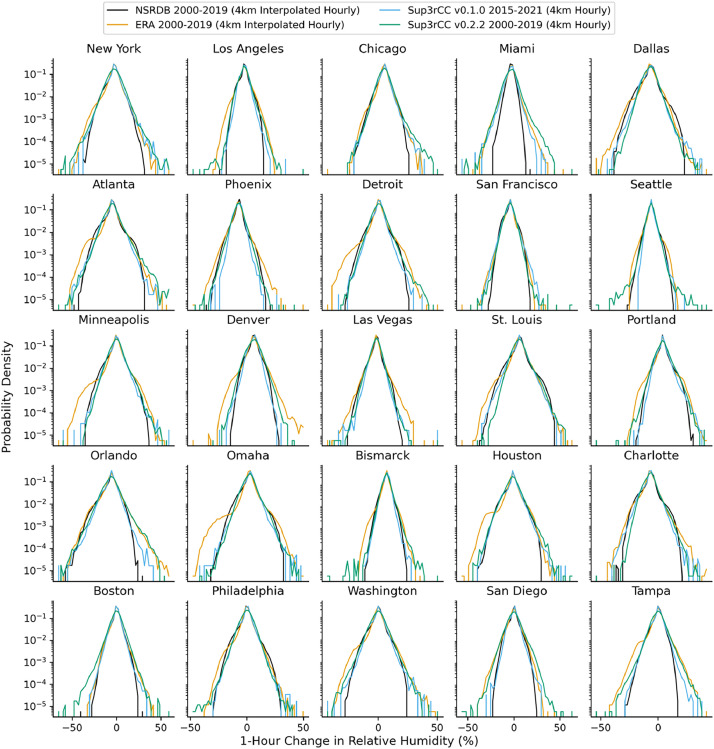
Probability distributions for 1-hour changes in relative humidity. Data are included for 25 of the largest cities in the contiguous United States comparing the NSRDB, ERA5, and Sup3rCC versions.

**Fig. 20:**
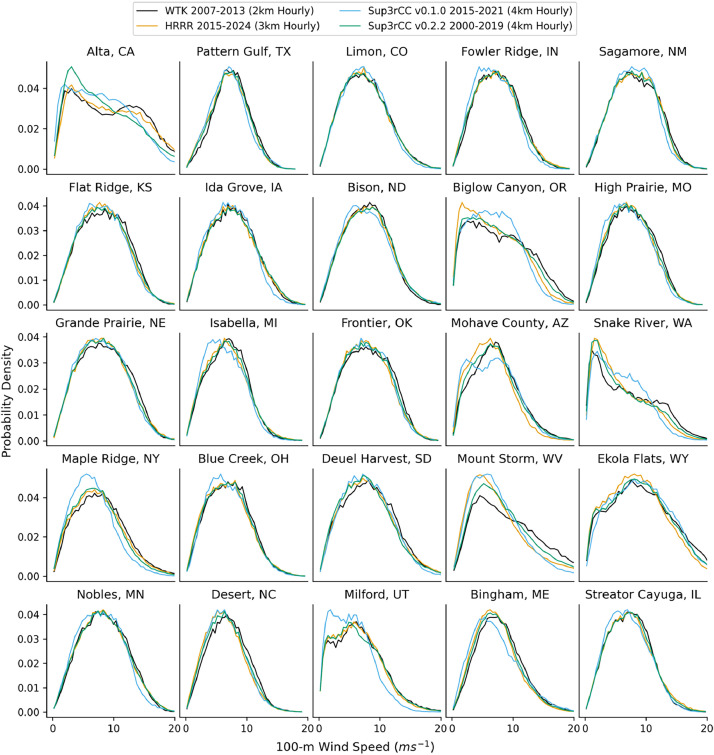
Probability distributions of hourly wind speeds. Data are included for 25 large wind energy facilities in the contiguous United States comparing the WTK, HRRR, and Sup3rCC versions.

**Fig. 21:**
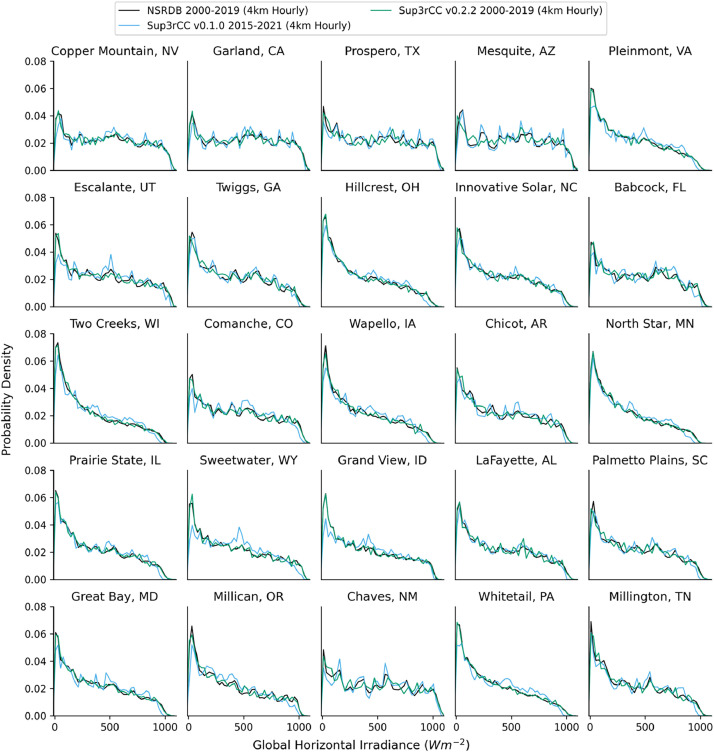
Probability distributions of hourly GHI. Data are included for 25 large solar energy facilities in the contiguous United States comparing the NSRDB and Sup3rCC versions. Nighttime hours are excluded.

**Fig. 22:**
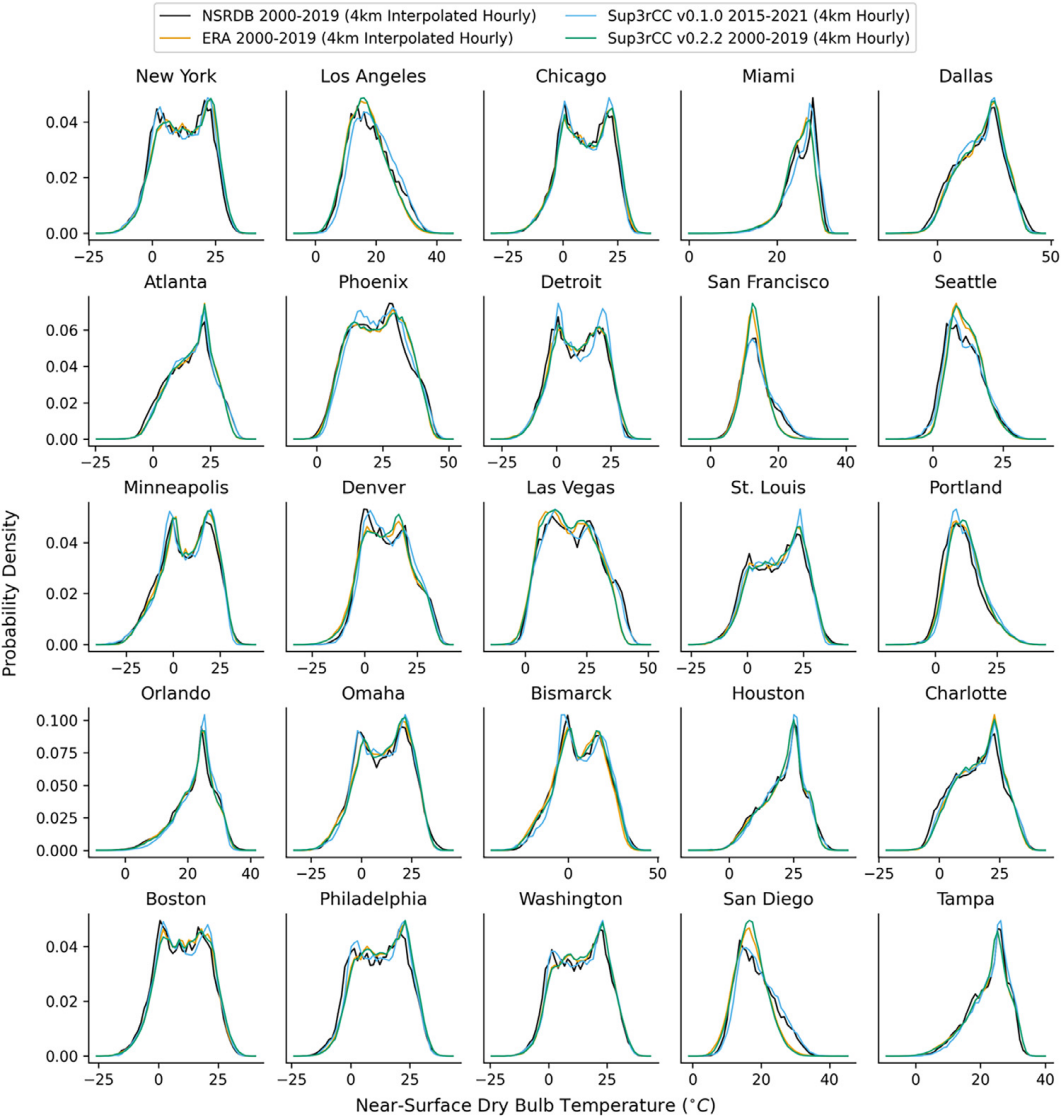
Probability distributions of hourly dry-bulb temperature. Data are included for 25 of the largest cities in the contiguous United States comparing the NSRDB, ERA5, and Sup3rCC versions.

**Fig. 23:**
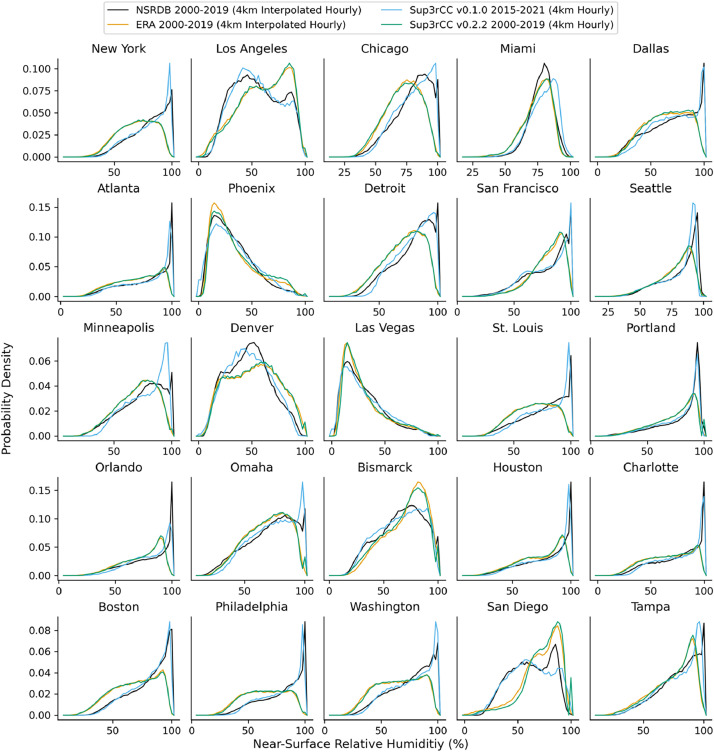
Probability distributions of hourly relative humidity. Data are included for 25 of the largest cities in the contiguous United States comparing the NSRDB, ERA5, and Sup3rCC versions.

**Fig. 24:**
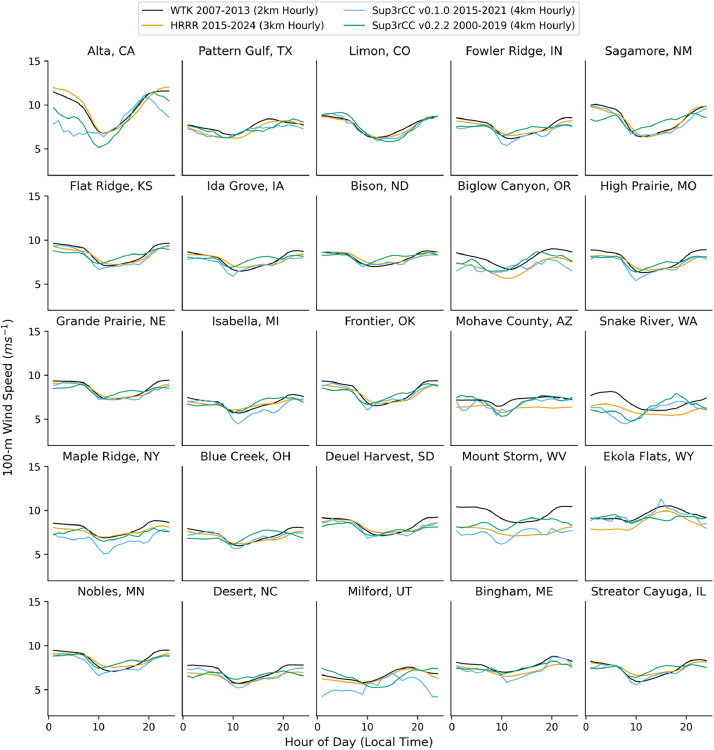
Mean diurnal cycles for 100-m wind speed. Data are included for 25 large wind energy facilities in the contiguous United States comparing the WTK, HRRR, and Sup3rCC versions.

**Fig. 25:**
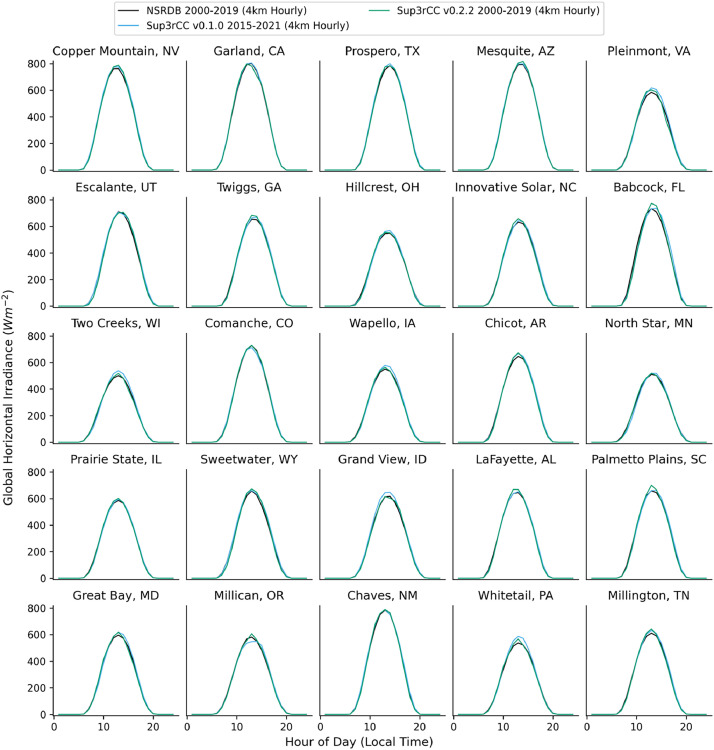
Mean diurnal cycles for hourly GHI. Data are included for 25 large solar energy facilities in the contiguous United States comparing the NSRDB and Sup3rCC versions.

**Fig. 26:**
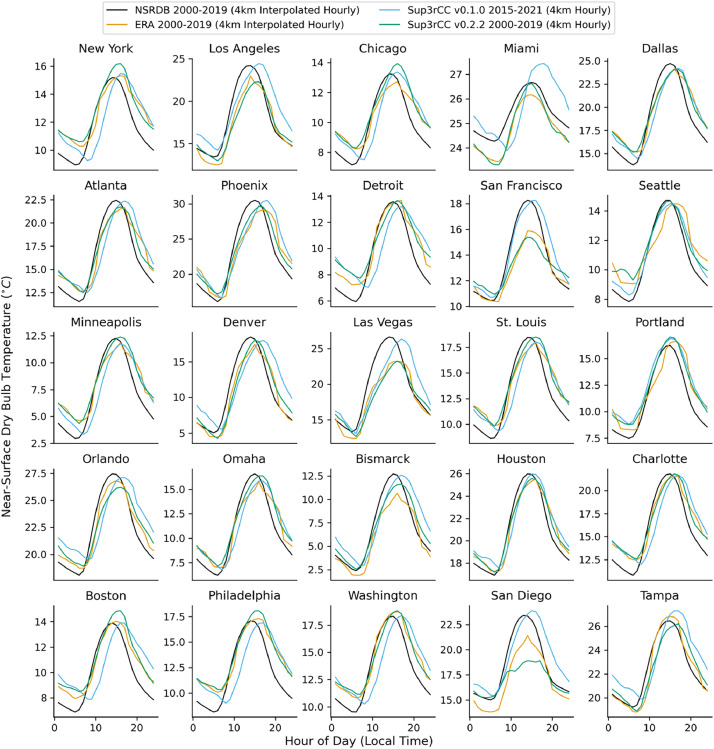
Mean diurnal cycles for hourly dry-bulb temperature. Data are included for 25 of the largest cities in the contiguous United States comparing the NSRDB, ERA5, and Sup3rCC versions.

**Fig. 27:**
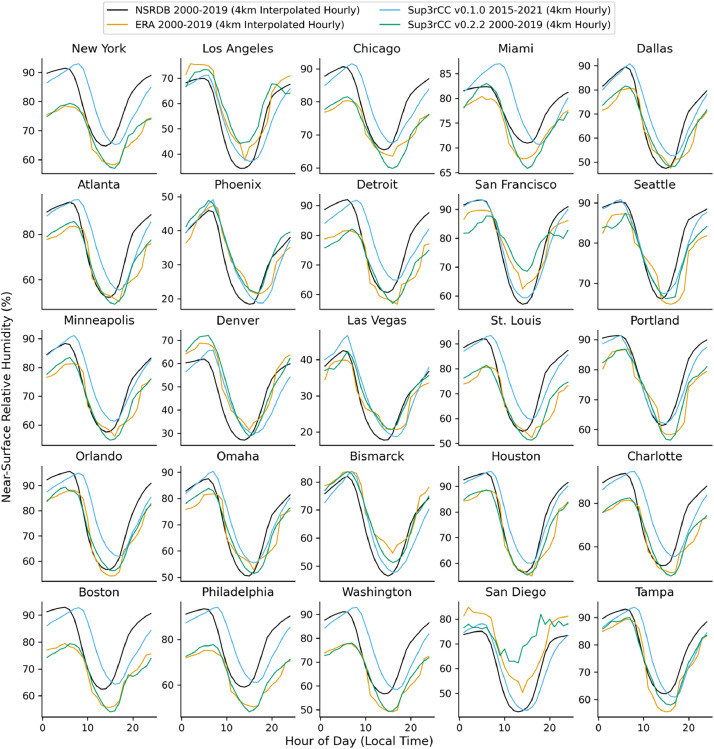
Mean diurnal cycles for hourly relative humidity. Data are included for 25 of the largest cities in the contiguous United States comparing the NSRDB, ERA5, and Sup3rCC versions.

**Fig. 28:**
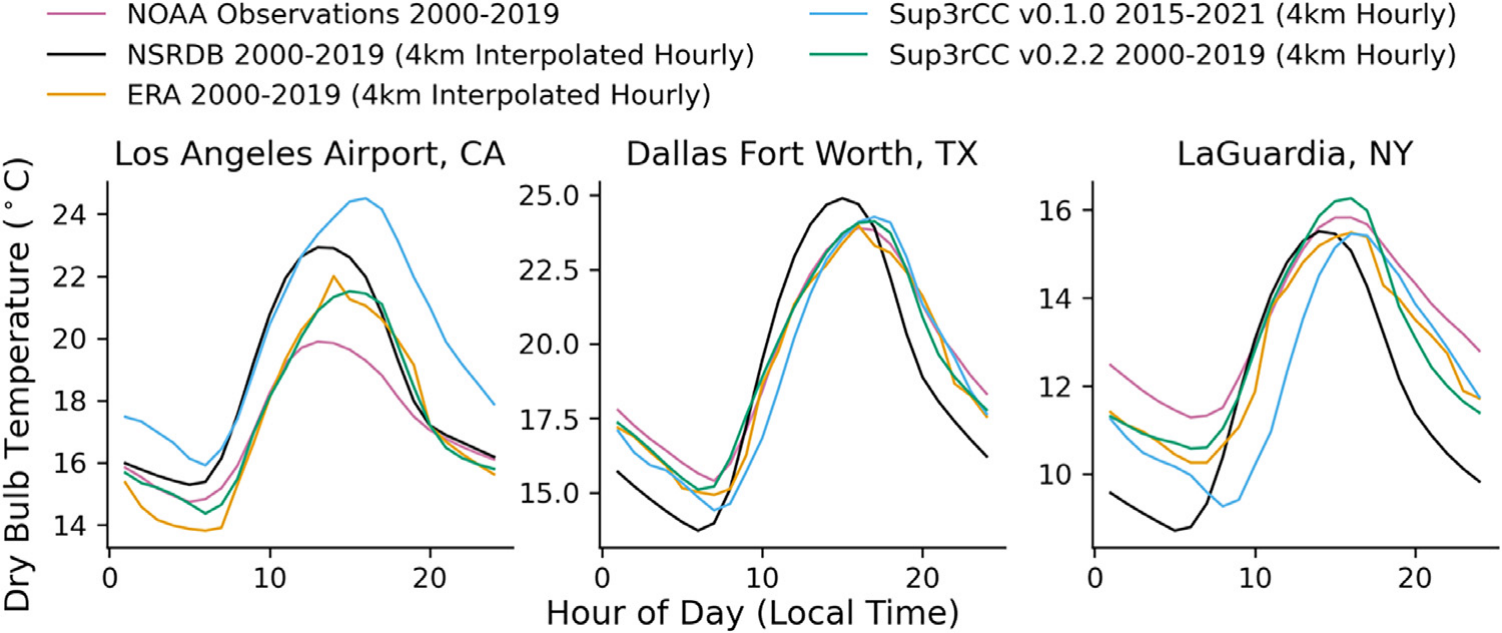
Mean diurnal cycles for modelled vs. observed hourly dry-bulb air temperature. Plots compare gridded datasets (e.g., NSRDB, ERA5, and Sup3rCC) to historical observation from the National Oceanic and Atmospheric Administration (NOAA) at three major airports.

**Fig. 29:**
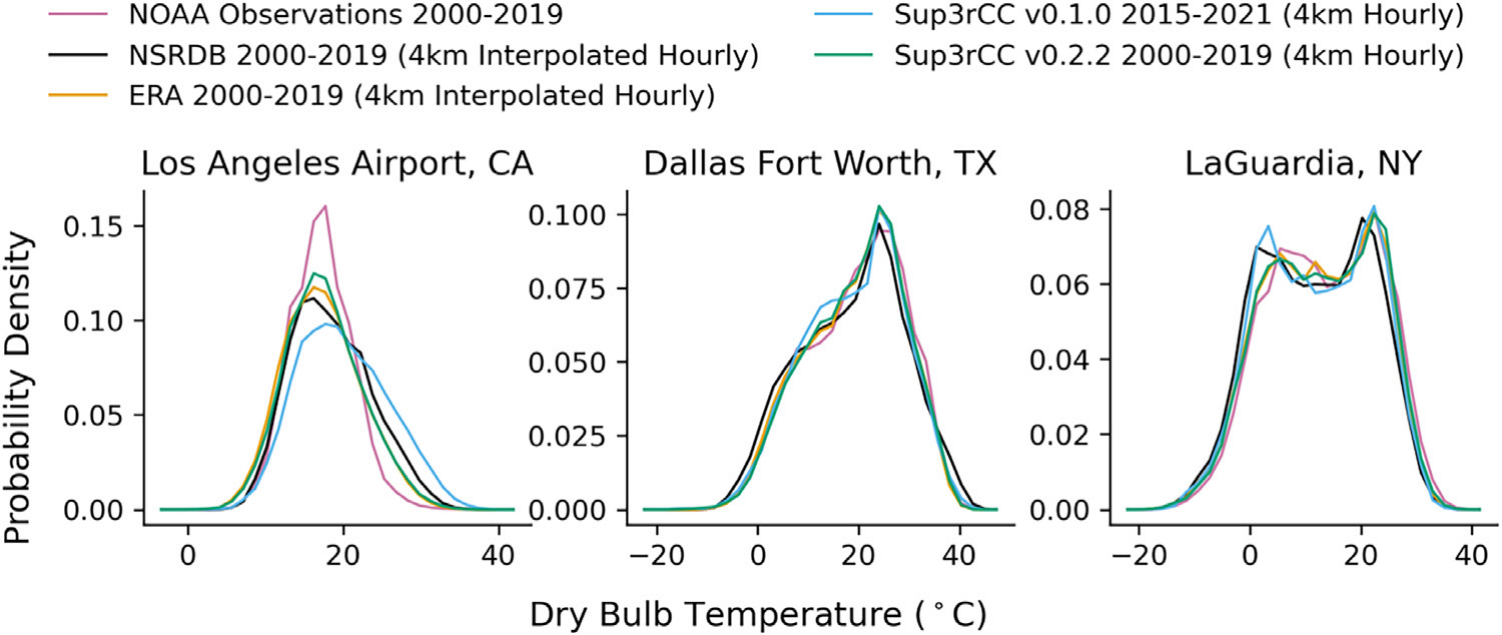
Probability distributions for modelled vs. observed hourly dry-bulb air temperature. Plots compare gridded datasets (e.g., NSRDB, ERA5, and Sup3rCC) to historical observation from NOAA at three major airports.

**Fig. 30:**
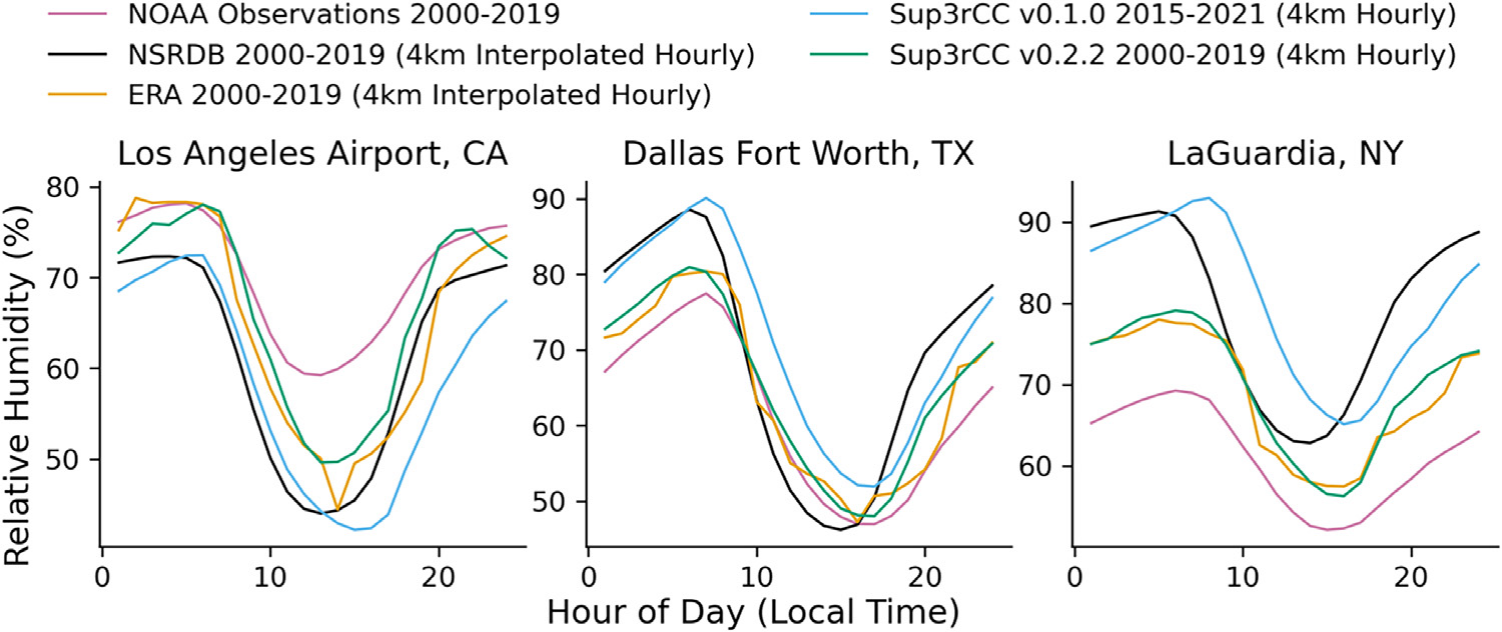
Mean diurnal cycles for modelled vs. observed hourly relative humidity. Plots compare gridded datasets (e.g., NSRDB, ERA5, and Sup3rCC) to historical observation from NOAA at three major airports.

**Fig. 31:**
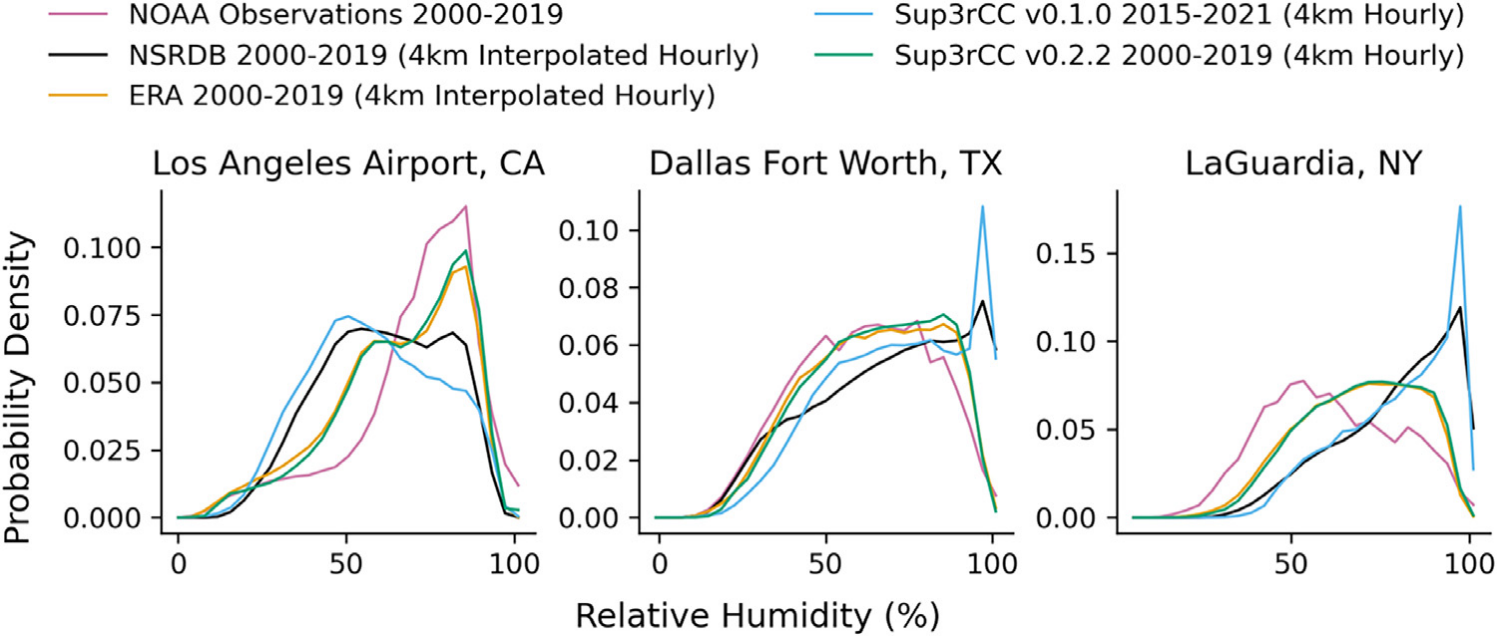
Probability distributions for modelled vs. observed hourly relative humidity. Plots compare gridded datasets (e.g., NSRDB, ERA5, and Sup3rCC) to historical observation from NOAA at three major airports.

**Fig. 32:**
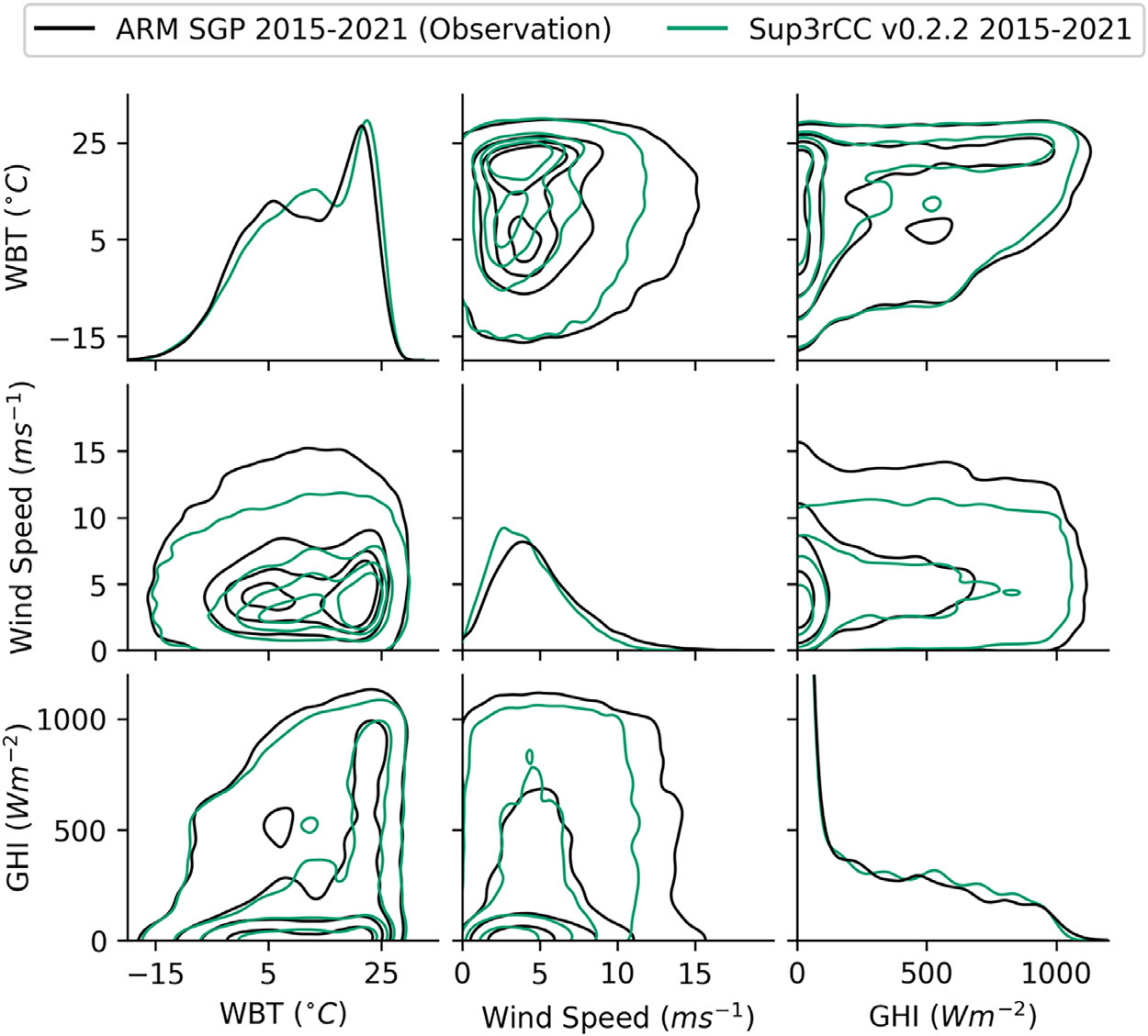
Joint distributions comparing ground measurements to hourly Sup3rCC v0.2.2 data for the current climate. Contour lines are iso-proportions of probability density that contain approximately 99%, 75%, 50%, and 25% of the probability mass from the outermost to innermost contour lines, respectively. Note the diagonal plots are univariate probability distributions and have y-axis units of probability density. Data were taken at the ARM SGP facility in Oklahoma. Note wet-bulb temperature (WBT) is used here as a composite representation of air temperature and humidity.

**Fig. 33:**
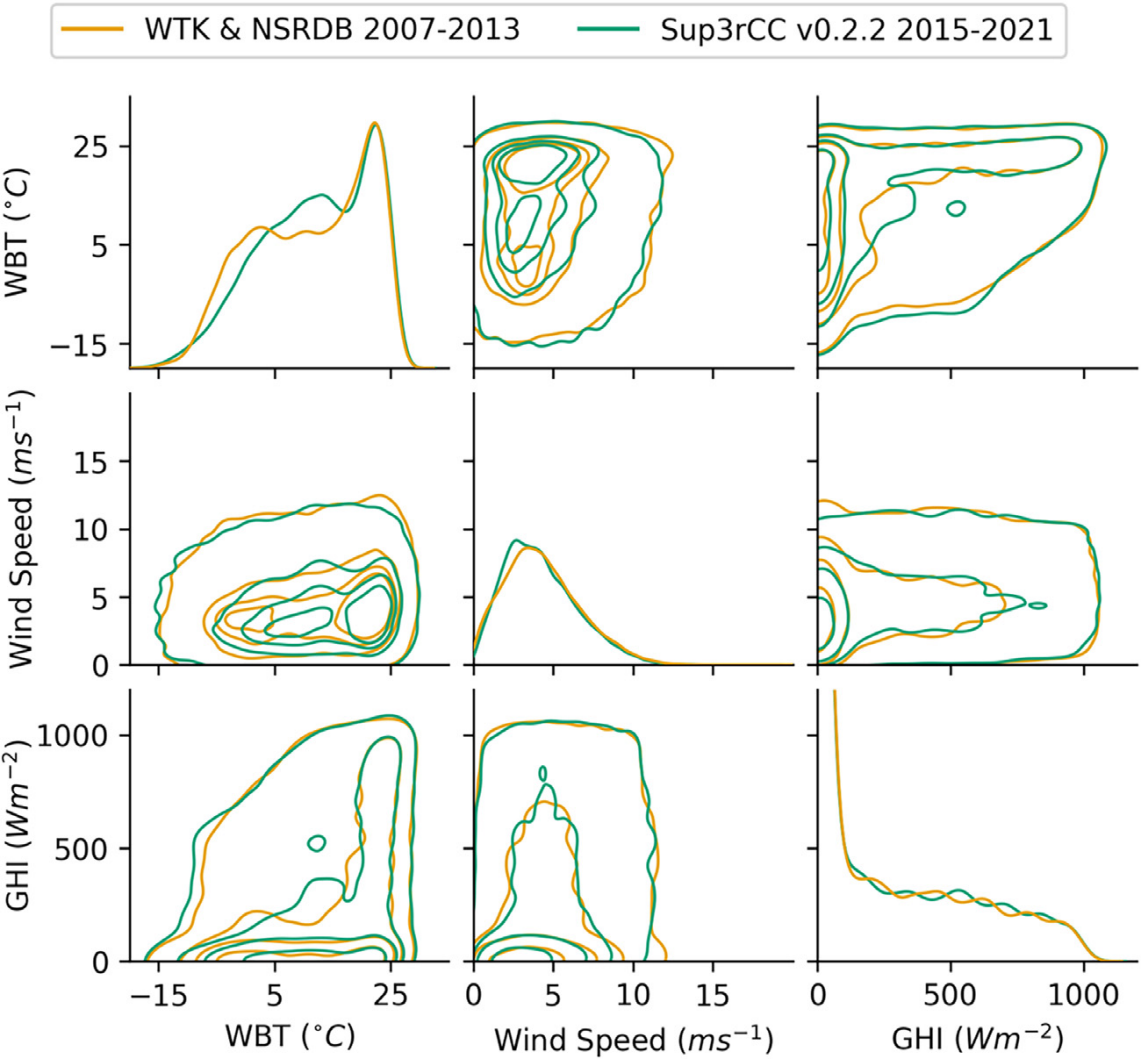
Joint distributions comparing WTK and NSRDB to hourly Sup3rCC v0.2.2 data for the current climate. Contour lines are iso-proportions of probability density that contain approximately 99%, 75%, 50%, and 25% of the probability mass from the outermost to innermost contour lines, respectively. Note the diagonal plots are univariate probability distributions and have y-axis units of probability density. Data were taken at the ARM SGP facility in Oklahoma. Note that WBT is used here as a composite representation of air temperature and humidity.

**Fig. 34:**
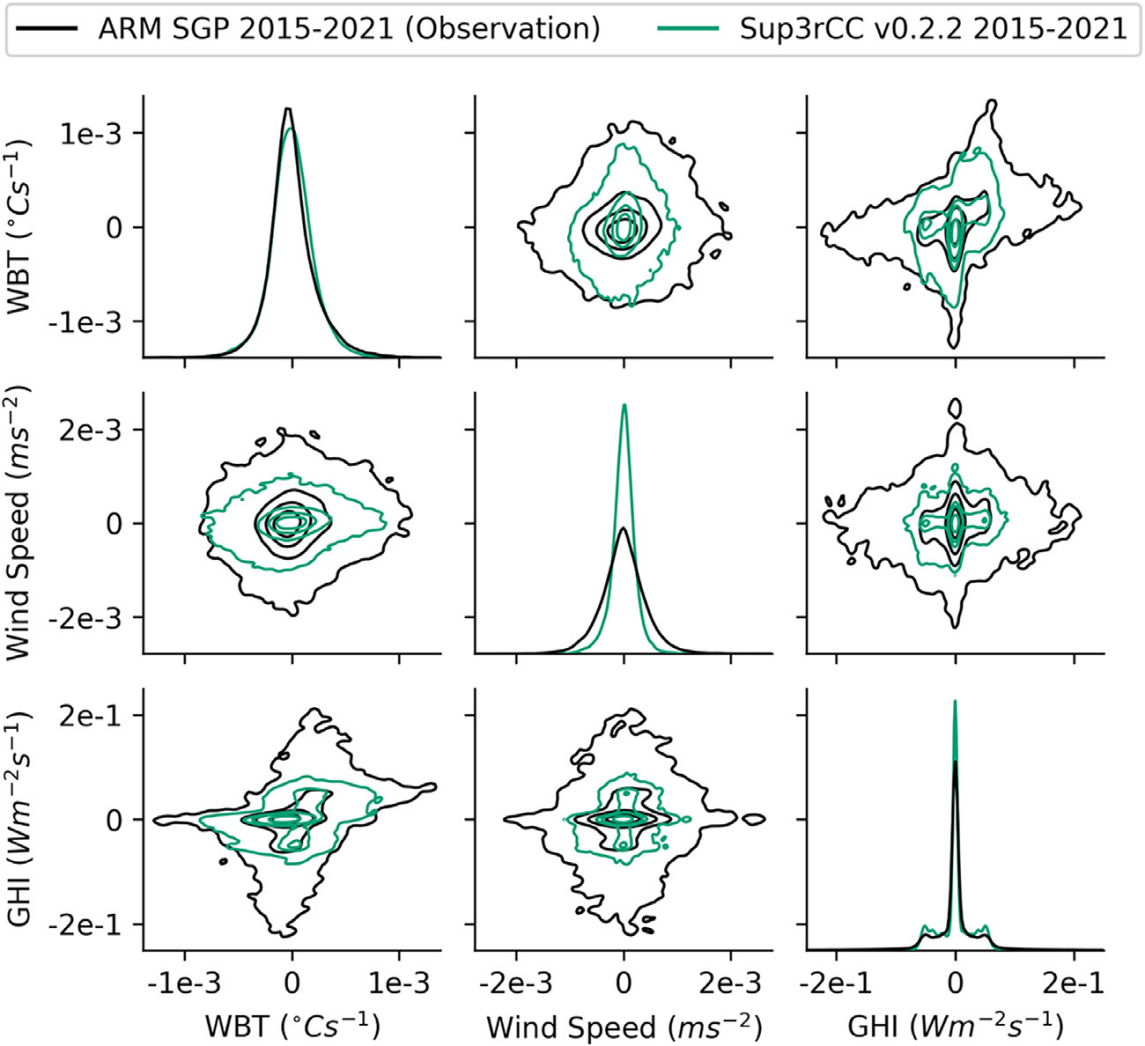
Joint distributions comparing time derivatives of ground measurements to Sup3rCC v0.2.2 data. Contour lines are iso-proportions of probability density that contain approximately 99%, 75%, 50%, and 25% of the probability mass from the outermost to innermost contour lines, respectively. Note the diagonal plots are univariate probability distributions and have y-axis units of probability density. Data were taken at the ARM SGP facility in Oklahoma. Note that WBT is used here as a composite representation of air temperature and humidity.

**Fig. 35:**
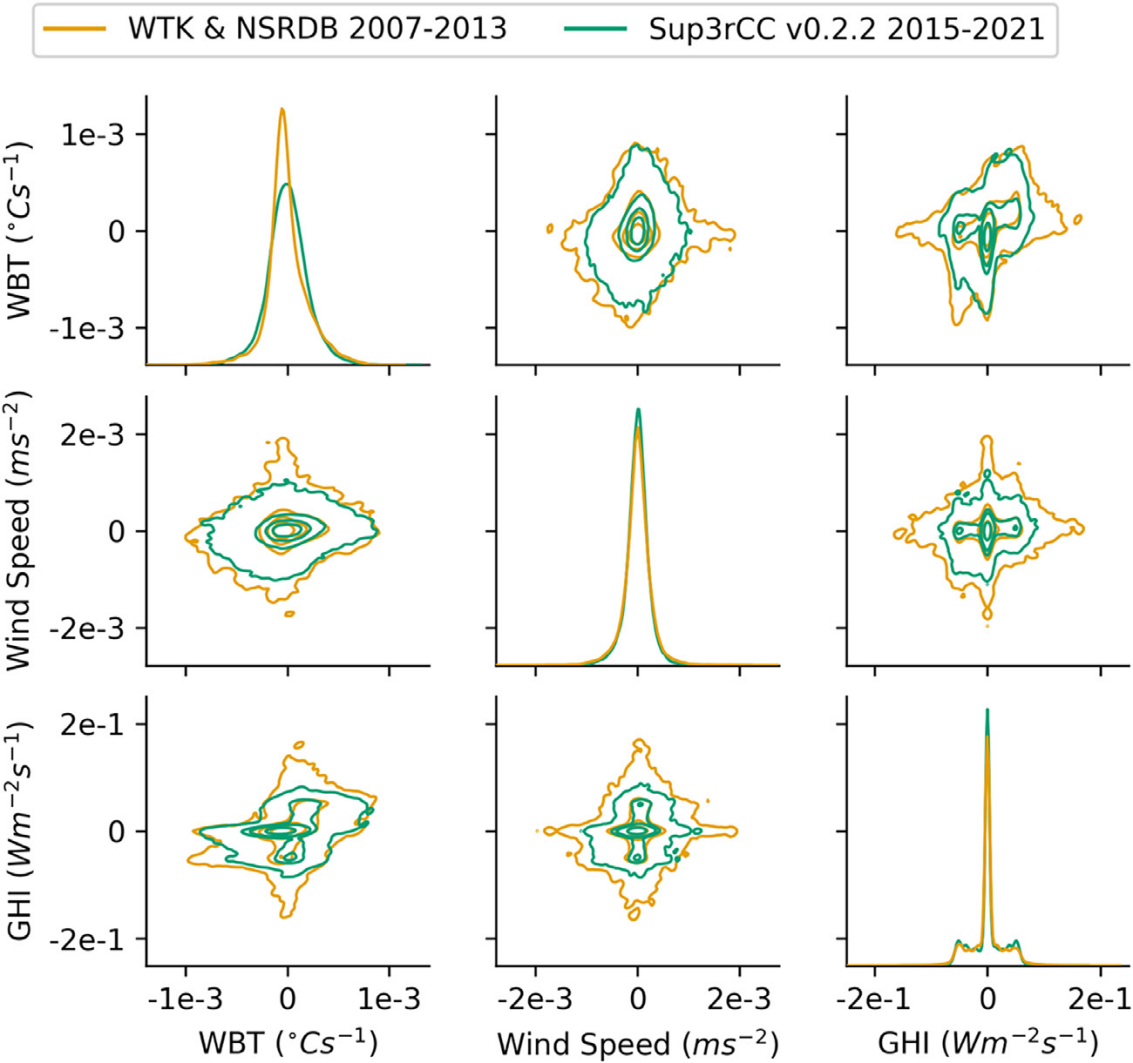
Joint distributions comparing time derivatives of WTK and NSRDB to Sup3rCC v0.2.2 data. Contour lines are iso-proportions of probability density that contain approximately 99%, 75%, 50%, and 25% of the probability mass from the outermost to innermost contour lines, respectively. Note the diagonal plots are univariate probability distributions and have y-axis units of probability density. Data were taken at the ARM SGP facility in Oklahoma. Note that WBT is used here as a composite representation of air temperature and humidity.

## 4 Experimental Design, Materials, and Methods

The second-generation Sup3rCC dataset builds on the original methods described in the original work [1] with a few minor updates. Notably, the data now undergo a double-bias correction at both 100-km and 4-km resolutions using nonlinear trend-preserving Quantile Delta Mapping (QDM) [14]; the data now include spatial downscaling for precipitation fields, and the generative models were retrained with a few general improvements. These three updates are further described in the following subsections. All software and pretrained models used to create these data can be found in the open-source repository on github [4] and the data repository on OEDI [13].

### 4.1 Double-bias correction

A key limitation of the original v0.1.0 Sup3rCC data was the simplistic low-resolution bias correction method and residual bias after downscaling compared to historical datasets [1].

First, the linear bias correction method was not trend-preserving. That is, if the ESM data were found to be under dispersed, variability was increased in the historical data using a multiplicative term. However, this increase would also exacerbate the signal of change represented in the ESM’s future projections, potentially even exacerbating extreme events in an unrealistic manner (illustrated in Fig. 11).

Second, the original v0.1.0 Sup3rCC data were generated from the SSP5-8.5 scenario starting in 2015, providing only 7 years of overlapping historical climate data for the historical bias correction. State-of-the-art downscaling research commonly uses multiple decades (up to 40 years [15]) to accurately quantify the historical climate, so the original Sup3rCC data were prone to misrepresentation of the historical climate by the bias present in the 7 selected years.

Finally, the original simplistic bias correction method did not actually reduce all the biases in the Sup3rCC data. There was still considerable residual bias in the original dataset at the 4-km resolution. This caused problems in integrating the data into power system models: Users could not be sure if differences in wind and solar resources could be attributed to projections of change in the ESMs or to historical biases. Additional steps were required to bias-correct the v0.1.0 Sup3rCC data before use so the power system modelling results were sensible.

To resolve these limitations, the new Sup3rCC v0.2.2 data use a double-bias correction method at both the 100-km and 4-km resolutions similar to other impacts assessments in the literature [15]. Bias correction is performed with QDM [14], which treats the different quantiles in a distribution explicitly (including extreme events) and preserves trends from the ESM. QDM corrects future model projections $x_{m,p}$ from the ESM at 100 km or Sup3rCC at 4 km with Eq. 1:

(1) $${\hat{x}}_{m,p}=F_{o,h}^{-1}\left(q_{m,p}\right)\times \frac{x_{m,p}}{F_{m,h}^{-1}\left(q_{m,p}\right)}$$

where ${\hat{x}}_{m,p}$ is the bias-corrected value, $q_{m,p}$ is the quantile of $x_{m,p}$ based on the statistics calculated from the future model projected data, $F_{o,h}^{-1}$ is the quantile function using the statistics calculated from the observed historical data, $F_{m,h}^{-1}$ is the quantile function using the statistics calculated from the historical model data. Eq. 1 preserves relative changes in meteorological variables. Eq. 2 preserves absolute changes:

(2) $${\hat{x}}_{m,p}=F_{o,h}^{-1}\left(q_{m,p}\right)+x_{m,p}-F_{m,h}^{-1}\left(q_{m,p}\right)$$

For historical ESM data, the projected data and historical data distributions are the same, so $F_{m,h}^{-1}\left(q_{m,p}\right)=x_{m,p}$ and the equation simplifies to basic quantile mapping ${\hat{x}}_{m,p}=F_{o,h}^{-1}\left(q_{m,p}\right)$.

All bias correction statistics are calculated and applied on a seasonal basis, with 24 distributions calculated per year and each calculation spanning 90 days. That is, data from early January are bias corrected based on data from January 1^st^ ±45 days across multiple decades. The only exception is precipitation data at the downscaled 4-km resolution, which were corrected using annual statistics (minor amounts of resulting seasonal bias can be seen in Fig. 3).

100-km daily-average ESM data from 1980 to 2014 in the historical scenario and from 2015 to 2019 in the SSP2-4.5 scenario were bias-corrected using a selection of high-quality 40-year historically accurate temperature, humidity, precipitation, and wind datasets from ERA5 [16] and Daymet [17].

Because there is no historically accurate 40-year 4-km hourly wind dataset commonly used in the energy research field, Sup3rCC wind data from 2000 to 2019 were corrected using a combination of the WTK [18] and HRRR [19] from 2007 to 2024 (excluding 2014 which is not present in either dataset). Solar irradiance data were corrected over 2000–2019 at both the 100-km daily-average and 4-km hourly resolutions using the NSRDB [20].

The 4-km hourly temperature and relative humidity are bias corrected using data from ERA5 interpolated to 4 km using lapse rates and elevation adjustments. This includes a step to minimize deviation from the source ERA5 data by correcting the low-resolution bias introduced by the lapse rate interpolation. This process is documented in the original Sup3rCC methods and associated open-source software [[1], [4]].

Surface pressure is treated differently because of its minor role in energy system modelling and its minor spatiotemporal variability compared to the other variables. Surface pressure is interpolated from un-bias-corrected ESM data (without a trained generative model; same as in the original work [1]) and is bias corrected at the 4-km hourly resolution using a simple additive term calculated from the multi-year-mean surface pressure in the WTK and HRRR.

Table 3 records the historical data sources and temporal extents used for the bias correction.

### 4.2 Spatially downscaled precipitation

We trained a new Sup3rCC model to generate 4-km daily average precipitation fields from 100-km daily ESM inputs. The intended use cases of these data include hydrological and hydropower modelling based on previous work using daily precipitation data, [15] so the Sup3rCC precipitation is output at a daily resolution unlike the outputs for wind, solar, temperature, and humidity. The model was trained on 4-km daily data from Daymet [17] using the same adversarial training procedure as the original Sup3rCC work [1]. The ESM data and the downscaled outputs were both bias corrected with QDM based on Daymet data at 100-km and 4-km resolutions.

The generative models struggled to reproduce the highly non-normal precipitation distributions with extreme precipitation values and low-precipitation drizzle days at 4 km so they were trained on the log-transform (log(x+1)) of the mm/day precipitation values, x. Log transforms are commonly used to improve heavy-tailed distributions for training with deep learning models, and this was found to improve the model’s ability to output precipitation values spanning several orders of magnitude as precipitation so often does. These transformed values are also mean-centered and divided by their standard deviation as is common practice in preparing data for training. The precipitation data were then transformed back to kg/m^2^s for the final Sup3rCC data product.

### 4.3 Improvements to the generative models

The original Sup3rCC modelling setup used up to three generative models per variable set to perform a 25x spatial enhancement in both horizontal dimensions and a 24x temporal enhancement in the temporal dimension for a 15,000x total increase in information content. This Sup3rCC generative modelling setup [1] built on earlier spatial downscaling work by adding a temporal enhancement model to an initial two-step spatial enhancement. Although this was a significant advancement for generative super resolution with temporal dynamics, it exhibited limitations in the effective receptive field (ERF) of the temporal dynamics model. With 38 convolutional layers of kernel size three and stride one, the dynamics model could pass information only across 38 horizontal grid cells from day to day. This limited the ability of the generative model to propagate strong wind fronts from day to day at the 4-km resolution, causing abnormal behavior at the daily interfaces.

A reorganization of the generative model stack with the temporal dynamics running first at 100-km resolution effectively increased the ERF in geographic space and resulted in improved large-scale dynamics. The original two-step spatial enhancement was found to be unnecessary and a single 25x spatial enhancement model with 3D convolutional layers (convolving across the spatial and temporal dimensions) was found to create realistic 4-km hourly fields from 100-km hourly inputs.

The new generative models also combine several near-surface variables (temperature, humidity, and wind) to improve the intervariable coherence for weather systems that acted on all at the same time such as a cold wave with high humidity and strong leading winds. The previous Sup3rCC data showed good intervariable coherence, but we found that this consolidation of variables into fewer generative models improves the synchronicity—especially at smaller scales. Ideally, all variables would be consolidated into a single model, but the remaining challenge of training a solar model across nighttime continues to prevent this.

The new models are shown to reproduce the previous v0.1.0 validation basis as shown in Fig. 13, Fig. 14, Fig. 15, Fig. 16, Fig. 17, Fig. 18, Fig. 19, Fig. 20, Fig. 21, Fig. 22, Fig. 23, Fig. 24, Fig. 25, Fig. 26, Fig. 27, Fig. 28, Fig. 29, Fig. 30, Fig. 31, Fig. 32, Fig. 33, Fig. 34, Fig. 35. They are still strongly conditioned on input fields with minimal deviation from ESM inputs even with warming through mid-century as shown in Fig. 11.

The new models perform with exceptional computational efficiency and can downscale 1 year of data over the contiguous United States to 1 million 4-km horizontal grid cells at an hourly frequency in just 24 node hours on a dual-socket Intel Xeon Sapphire Rapids 52-core processor (104 cores total) with 256 gigabytes of memory. This is 7x faster than the original Sup3rCC work and 283x faster than the original compute estimate for a comparable numerical weather prediction model [1].

## Limitations

The new v0.2.2 Sup3rCC data share similar limitations to the original v0.1.0 Sup3rCC data. For example, Sup3rCC will not produce many phenomena that are not present in ESM data (e.g., hurricanes, tornadoes, wildfires, and convective storms). Sup3rCC still has a relatively small number of output variables at few elevations above surface compared to a numerical weather model product, limiting the potential applications of the data. Note the generative models have difficulty in reproducing some historical weather attributes such as subtle wind diurnal cycles (Fig. 24) and cloud transport, leading to less frequent large solar ramps (Fig. 17).

It should be noted the data are representative only of *possible* weather; the historical Sup3rCC years represent the historical climate and will not accurately reproduce real historical weather events. Similarly, the future weather projections are intended to represent possible meteorological futures based on the latest earth system science from CMIP6 with uncertainty represented in the various ESM and SSP scenarios, but these projections are in no way a guarantee of future meteorological conditions.

We release these data based on the validation presented here and previous methodological documentation [1] for applications in energy system analyses, renewable energy resource assessments, system planning activities, and grid resilience studies. Additional applications of the data may be appropriate, but users are cautioned to first understand the validation basis and limitations of future meteorological projections before relying on the data for decision making.

## Ethics Statement

The authors have read and follow the ethical requirements for publication in Data in Brief and confirm that this work does not involve human subjects, animal experiments, or any data collected from social media platforms.

## CRediT authorship contribution statement

**Grant Buster:** Conceptualization, Investigation, Methodology, Software, Validation, Visualization, Writing – original draft, Writing – review & editing, Project administration, Funding acquisition. **Brandon N. Benton:** Conceptualization, Software, Writing – original draft, Writing – review & editing. **Deeksha Rastogi:** Conceptualization, Data curation, Visualization, Validation. **Shih-Chieh Kao:** Conceptualization, Data curation, Validation, Funding acquisition. **Guilherme Castelao:** Investigation, Software. **Jordan Eisenman:** Investigation, Data curation.

## Data Availability

OEDISuper-Resolution for Renewable Energy Resource Data with Climate Change Impacts (Sup3rCC) (Original data).
